# Stomach secretes estrogen in response to the blood triglyceride levels

**DOI:** 10.1038/s42003-021-02901-9

**Published:** 2021-12-07

**Authors:** Takao Ito, Yuta Yamamoto, Naoko Yamagishi, Yoshimitsu Kanai

**Affiliations:** Cell Biology and Anatomy, Graduate School of Medicine, Wakayama Medical University, Wakayama, Japan

**Keywords:** Fat metabolism, Steroid hormones, Fats, Gastrointestinal hormones, Metabolic syndrome

## Abstract

Mammals receive body energy information to maintain energy homeostasis. Ghrelin, insulin, leptin and vagal afferents transmit the status of fasting, blood glucose, body fat, and food intake, respectively. Estrogen also inhibits feeding behavior and lipogenesis, but increases body fat mass. However, how blood triglyceride levels are monitored and the physiological roles of estrogen from the perspective of lipid homeostasis remain unsettled. Here, we show that stomach secretes estrogen in response to the blood triglyceride levels. Estrogen-secreting gastric parietal cells predominantly use fatty acids as an energy source. Blood estrogen levels increase as blood triglyceride levels rise in a stomach-dependent manner. Estrogen levels in stomach tissues increase as blood triglyceride levels rise, and isolated gastric gland epithelium produces estrogen in a fatty acid-dependent manner. We therefore propose that stomach monitors and controls blood triglyceride levels using estrogen, which inhibits feeding behavior and lipogenesis, and promotes triglyceride uptake by adipocytes.

## Abstract (web summary)

Ito et al. demonstrate that murine gastric parietal cells produce estrogen using fatty acids as an energy source, and secrete estrogen in response to the blood triglyceride levels. The authors propose that the stomach is involved in the monitoring and the regulation of blood triglyceride levels.

## Introduction

Maintaining energy homeostasis is critical for mammals to survive. The central nervous system receives information about the body’s energy status to control feeding behavior, lipogenesis, and so on^1,2^. Several mechanisms for sensing and informing the body’s energy status have been clarified, including fasting (ghrelin, from the stomach), blood glucose (insulin and glucagon, from the pancreas), body fat (leptin, from adipose tissues), and ingested nutrients (vagus nerves and gut-secreted peptides, e.g., glucagon-like peptide 1 [GLP1], gastric inhibitory peptide (GIP), cholecystokinin [CCK], and peptide YY [PYY], from the intestines and the liver) levels^2–6^. Surprisingly, however, the hormones or nerves that sense and inform the blood triglyceride levels, such as insulin for the blood glucose levels, have not been reported.

**Fig. 1:**
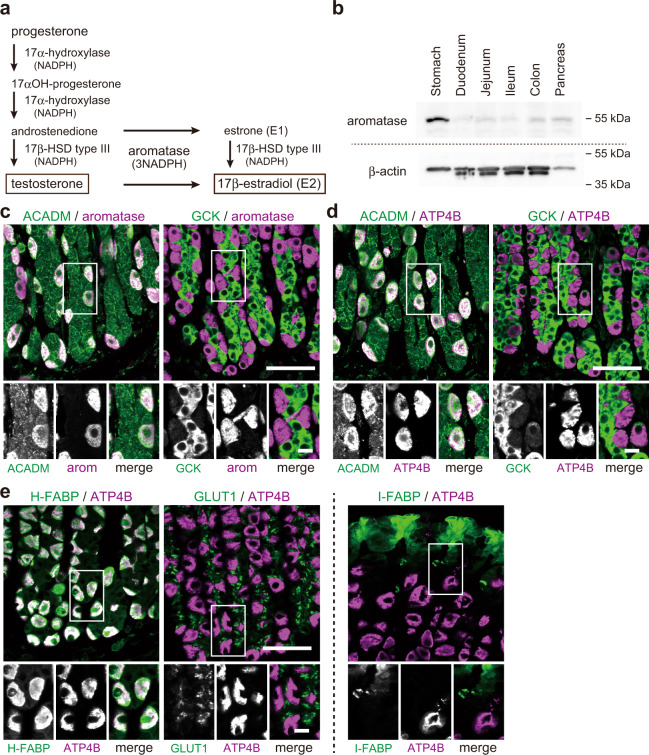
Estrogen-producing gastric parietal cells use fatty acids as an energy source in males. **a** Scheme of testosterone (progesterone)-17β-estradiol (E2) conversion pathway in rat gastric parietal cells. **b** Immunoblotting of crude extracts from the stomach, duodenum, jejunum, ileum, colon, and pancreas of adult male rats using antibodies against aromatase and control β-actin. **c**–**e** Gastric mucosa from male rat was double-stained for ACADM (acyl-CoA dehydrogenase medium-chain, an enzyme involved in β-oxidation) or GCK (glucokinase, a glycolytic enzyme) (green) with aromatase [arom] (magenta) (**c**), for ACADM or GCK (green) with ATP4B (H + /K + ATPase, a gastric parietal cell marker; magenta) (**d**), and for H-FABP (heart-type fatty acid-binding protein [FABP]), GLUT1 (glucose transporter 1), or I-FABP (intestinal FABP) (green) with ATP4B (magenta) (**e**). Bars: (main panels) 50 µm; (insets) 10 µm.

Estrogen is a multi-target and multi-functional hormone beyond just a sex hormone; it controls glucose/lipid homeostasis, bone metabolism, brain function, and skeletal growth as well as follicular growth and ovulation^7^. Besides the ovaries in females, estrogen is also secreted from adipose tissues, which is greatly increased in males^8,9^. Our previous studies showed that parietal cells in rat stomach also express enzymes for the production of estrogens, such as aromatase, together with 17α-hydroxylase and 17β-hydroxysteroid dehydrogenase type III (17β-HSD type III), and convert testosterone or progesterone to estrogen (17β-estradiol [E2])^10,11^ (Fig. 1a). Aromatase was prominently expressed in the stomach among the tissues upstream of the portal vein (stomach, duodenum, jejunum, ileum, colon, and pancreas)^10^, and the small amounts of aromatase detected in the rest of the tissues were mainly from the attached adipose tissues (Fig. 1b and Supplementary Fig. 1). Parietal cells secrete estrogen (gastric estrogen) into the portal vein, so estrogen levels are higher in the portal vein (~x2) than in the artery in male and female rats, which is lost in gastrectomized (GX) rats^10,12^. However, little is known about the role of gastric estrogen and how the secretion of gastric estrogen is regulated^13^.

Here, we show that parietal cells act as a sensor for the blood triglyceride levels and secrete gastric estrogen in response to the blood triglyceride levels, using male and ovariectomized (OVX) female rats. Estrogen inhibits the feeding behavior and de novo lipogenesis in the liver and adipocytes (decrease of lipid supply), but increases the white adipose tissue (WAT) mass under conditions of suppressed lipogenesis (an increase of lipid uptake by WAT)^14–19^ and the lipid consumption in muscles^20^. Therefore, we propose a model that stomach secretes estrogen to lower the blood triglyceride levels when they are high.

## Results

### Estrogen-producing gastric parietal cells use fatty acids as an energy source in males

Gastric estrogen directly enters the liver via the portal vein, where estrogen controls glucose/lipid homeostasis^15^. This secretion is similar to that of insulin, which is secreted from pancreatic β-cells, enters the portal vein, and directly acts on the liver to keep appropriate blood glucose levels^21,22^. β-cells, which sense the blood glucose levels, predominantly use glucose^23^ (Supplementary Fig. 1b), and parietal cells require energy (NADPH) in the production of estrogen (e.g., 3 x NADPH from testosterone, Fig. 1a). We, therefore, focused on the energy sources of parietal cells to obtain clues on how the production of gastric estrogen is regulated. Blood estrogen levels change in the menstrual cycle in females, so in this study, we used 8-week-old male and OVX female rats to exclude the effect of estrogen from the ovaries.

Triglyceride (fatty acids) and glucose are major sources of energy for mammals, so we examined the expression of the enzymes used in the energy generation from fatty acids (acyl-CoA dehydrogenase medium chain [ACADM], an enzyme involved in β-oxidation) and glucose (glucokinase [GCK], a glycolytic enzyme) in gastric mucosa of male rats (Fig. 1c, d). We identified the estrogen-producing cells and parietal cells using the antibodies against aromatase and H + /K + ATPase (ATP4B), respectively^10^. Aromatase-positive cells expressed high levels of ACADM, but little if any GCK. Conversely, aromatase-negative cells showed strong GCK but weak ACADM expressions (Fig. 1c). ATP4B-positive parietal cells showed the same result as the aromatase-positive cells (Fig. 1d). These indicate that parietal cells mainly use fatty acids in the production of estrogen. Fatty acid-binding proteins (FABPs) and glucose transporters (GLUTs) play important roles in the uptake or use of fatty acids and glucose, respectively, in a subtype-specific manner^24–26^, and parietal cells express heart-type FABP (H-FABP), but not intestine type one (I-FABP)^27^. For further confirmation of the dominant use of fatty acids in parietal cells, we examined the distribution of H-FABP and GLUT1, the most widely expressed GLUT^26^, in the stomach. Parietal cells were confirmed to express H-FABP, but not GLUT1 (Fig. 1e). We also examined the expression of I-FABP as a control FABP, but it was not expressed in parietal cells as reported. Our data conclude that parietal cells, which exclusively produce estrogen in the stomach, predominantly use fatty acids as an energy source in males.

### Blood estrogen levels increase as blood triglyceride levels rise in males

**Fig. 2:**
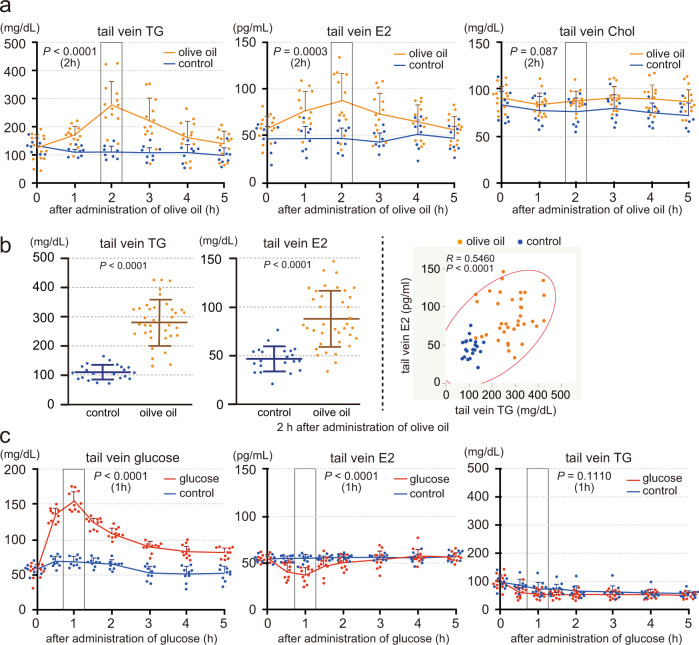
Blood estrogen levels increase as blood triglyceride levels rise in males. **a** and **b** Male rats (8 weeks old, deprived of food for 4 h) were orally administered olive oil (2.5 mL per kg body weight) or control water (control) using the gavage technique. Triglyceride (TG), E2 and cholesterol (Chol) levels in the tail venous blood were measured before (0) and at 1, 2, 3, 4, 5 h after the administration (olive oil: *n* = 13, control: *n* = 8, **a**). TG and E2 levels in the tail venous blood were measured at 2 h after the administration (olive oil: *n* = 37, control: *n* = 21, **b**, left), and the correlation diagram between the blood TG and E2 levels (*n* = 58, **b**, right). **c** Male rats (8 weeks old, deprived of food for 18 h) were orally administered glucose (2 g per kg body weight, *n* = 10) or control water (*n* = 9). Glucose, E2, and TG in the tail venous blood were measured before (0) and at 0.5, 1, 1.5, 2, 3, 4, and 5 h after the administration. *n* number of rats. Data were mean ± s.d. *P* values determined by two-sided Student’s *t*-test at 2 h (**a**, **b**, left) or 1 h (**c**) after the administration. **b**, right, *R* and *P* values determined by Pearson’s product-moment correlation with 95% density ellipse.

Given that parietal cells produce estrogen using fatty acids as an energy source, blood triglyceride levels could affect the secretion of gastric estrogen. We then investigated the relationship between the blood triglyceride and estrogen levels using male rats (Fig. 2). Triglyceride (olive oil, 2.5 mL per kg body weight) or control water was orally administered to male rats, and their tail venous triglyceride, estrogen (E2), and cholesterol levels were measured before (0 h) and at 1, 2, 3, 4, and 5 h after the administration (Fig. 2a). Blood triglyceride levels (basal: ~130 mg/dL) increased, peaked at 2 h post-administration (~280 mg/dL), and then declined to the basal levels (5 h) as reported previously^28^. Blood E2 levels (basal: ~55 pg/mL) also increased, peaked at 2 h after the administration (~85 pg/mL), and then returned to the basal levels, while blood cholesterol levels increased but were not significant. We then analyzed the relationship between the blood triglyceride and E2 levels at 2 h after the administration; there was a positive correlation between them (Fig. 2b).

We further examined the effect of blood glucose levels on the blood estrogen levels. Glucose administration decreases blood fatty acid levels via insulin-mediated inhibition of lipolysis in adipose tissues^29–31^, so we also monitored the blood triglyceride levels. Male rats were orally administered glucose (2 g per kg body weight) or control water, and their tail venous glucose, E2, and triglyceride levels were measured before (0 h) and at 0.5, 1, 1.5, 2, 3, 4, and 5 h after the administration (Fig. 2c). Blood glucose levels (basal: ~70 mg/dL) increased, peaked at 1 h after the administration (~170 mg/dL), and declined toward the basal levels^32^; however, blood E2 or triglyceride levels did not increase, rather decreased, when blood glucose levels were high. These data indicate that blood estrogen levels increase as blood triglyceride levels, but not glucose levels, rise in males.

### Stomach secretes estrogen in response to the blood triglyceride levels in males

**Fig. 3:**
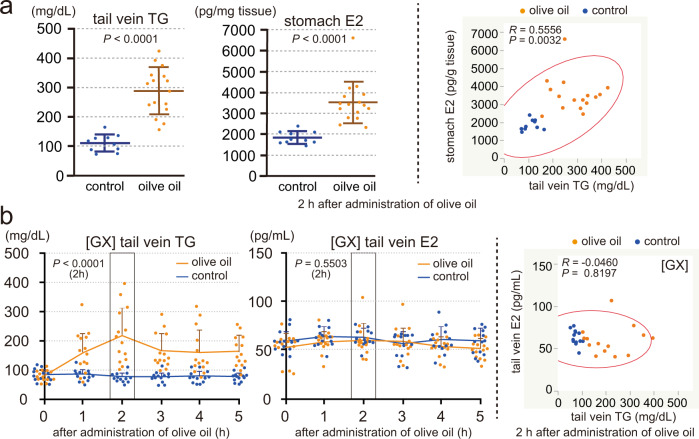
Stomach secretes estrogen in response to the blood triglyceride levels in males. **a** Male rats (8 weeks old, deprived of food for 4 h) were orally administered olive oil (2.5 mL per kg body weight, *n* = 16) or control water (*n* = 10). TG levels in the tail venous blood and E2 levels in the stomach tissues were measured at 2 h after the administration (left), and the correlation diagram between the blood TG and stomach E2 levels (*n* = 26, right). **b** GX male rats (8 weeks old, operated 5 days before, deprived of food for 4 h) were orally administered olive oil (5 mL per kg body weight, *n* = 14) or control water (*n* = 13). TG and E2 levels in the tail venous blood measured before (0) and at 1, 2, 3, 4, and 5 h after the administration (left), and the correlation diagram between the blood TG and E2 levels at 2 h after the administration (*n* = 27, right). *n* number of rats. Data were mean ± s.d. **a** left and **b** left, *P* values determined by two-sided Student’s *t*-test at 2 h after the administration. **a** right and **b** right, *R* and *P* values determined by Pearson’s product-moment correlation with 95% density ellipse.

Next, to investigate the relationship between the blood triglyceride and stomach E2 levels, tail venous triglyceride and stomach tissue E2 levels were measured at 2 h after the administration of olive oil or control water (Fig. 3a). Olive oil administered male rats showed elevated blood triglyceride (olive oil: ~280 mg/dL, control: ~100 mg/dL) and stomach E2 (olive oil: ~3,500 pg/g tissue, control: ~1900 pg/g tissue) levels, and there was a positive correlation between the blood triglyceride and stomach tissue E2 levels. These data indicate that the stomach produces estrogen in response to the blood triglyceride levels.

To confirm the roles of the stomach in the blood triglyceride level-dependent changes of the blood estrogen levels, we generated GX male rats to perform the olive oil administration study in the absence of stomach (Fig. 3b). GX rats showed a weaker increase of the blood triglyceride levels than normal rats when administered olive oil, so we used 5 mL per kg body weight of olive oil in this study. Five days after total gastrectomy, GX rats were administered olive oil or control water, and tail venous triglyceride and E2 levels were measured before (0 h) and at 1, 2, 3, 4, and 5 h after the administration (Fig. 3b, left). Blood triglyceride levels of GX rats (basal: ~90 mg/dL) increased, peaked at 2 h post-administration (~210 mg/dL), and then declined toward the basal levels; but, blood E2 levels did not show apparent changes (~60 pg/mL). We analyzed the relationship between the blood triglyceride and E2 levels at 2 h after the administration but found no correlation between them (Fig. 3b, right). These data indicate that the stomach is the responsible organ for the control of the blood estrogen levels in response to the blood triglyceride levels in males.

### Blood estrogen levels and production of gastric estrogen are directly regulated by blood triglyceride levels in males

**Fig. 4:**
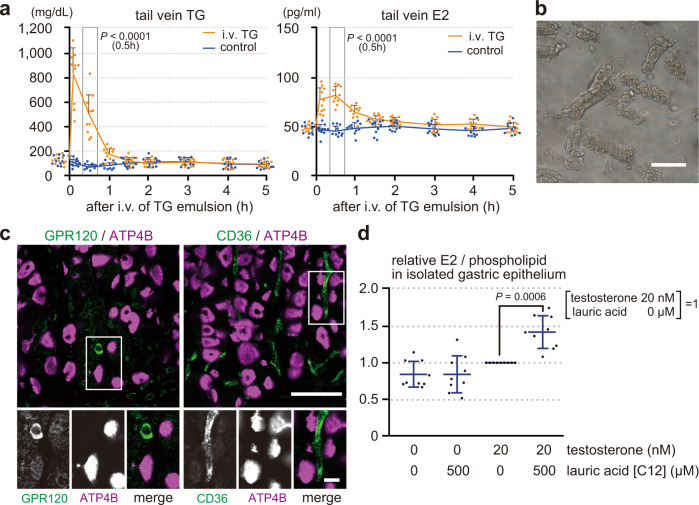
Blood estrogen levels and production of gastric estrogen are directly regulated by blood triglyceride levels in males. **a** Male rats (8 weeks old, deprived of food for 4 h) were intravenously injected with soy oil emulsion (2 mL of 20 % soy oil emulsion per kg body weight, *n* = 10) or control saline (control, *n* = 10). TG and E2 levels in the tail venous blood were measured before (0) and at 0.1, 0.5, 1, 1.5, 2, 3, 4, and 5 h after the injection. **b** Isolated gastric gland epithelium from male rats. **c** Gastric mucosa of the male rat was double-stained for GPR120 (G-protein-coupled receptor 120) or CD36 (cluster of differentiation 36) (green) with ATP4B (magenta). **d** Isolated epithelium from male rat (deprived of food for 4 h) was incubated in DMEM with or without testosterone (20 nM) or lauric acid (500 µM) at 37 °C for 1 h. E2 levels, normalized by phospholipid, were compared to those incubated with 20 nM testosterone but without lauric acid (*n* = 9). *n* number of rats. Data were mean ± s.d. **a** (0.5 h) and **d**, *P* values determined by two-sided Student’s *t*-test. Bars: (main panels) 50 µm; (insets) 10 µm.

Orally administered olive oil is lipolytic to fatty acids in the intestines. Fatty acids in the gastrointestinal tract play important roles in lipid homeostasis through fatty acid receptors (e.g., GPR120 and CD36)^33,34^, gastrointestinal hormones (e.g., CCK, GLP1, PYY, and Ghrelin)^3–5^, and nerves (e.g., vagal afferents)^2,6^. To increase the blood triglyceride levels without the effects of ingested fatty acids on the gastrointestinal tract, soy oil emulsion (2 mL of 20% soy oil emulsion per kg body weight) or control saline was injected into rat tail vein (Fig. 4a)^35,36^. Tail venous triglyceride and E2 levels were measured before (0 h) and at 0.1, 0.5, 1, 1.5, 2, 3, 4, and 5 h after the injection. Blood triglyceride levels peaked just after the injection and returned to the basal levels at 1–1.5 h after the injection (basal: ~150 mg/dL, peak: ~800 mg/dL). Blood E2 levels also increased rapidly and returned to the basal levels at 1.5–2 h after the injection (basal: ~50 pg/ml, peak: ~85 pg/ml). These data indicate that blood estrogen levels increase as blood triglyceride levels rise, even in the absence of the stimulation of the gastrointestinal tract by ingested fatty acids.

We further designed in vitro studies to avoid the possible indirect effects of fatty acids on parietal cells from other cells using isolated gastric gland epithelium (Fig. 4b). However, the stomach is reported to express fatty acid receptors, such as GPR120 and CD36^37^. Indeed, although these receptors were not detected in parietal cells, they were expressed in other types of cells in the gastric mucosa (Fig. 4c). On the other hand, parietal cells dominantly express H-FABP (Fig. 1e), which plays important role in the cellular uptake of fatty acids and their subsequent transport toward the β-oxidation system^38^. H-FABP binds to the fatty acids of C12 to 20, whereas GPR120 and CD36 to those of C14 or above and C20 or above, respectively^39–41^. These indicate that lauric acid (C12 fatty acid) can be used as an energy source in parietal cells without stimulating GPR120 or CD36 positive cells. Estrogen is converted from testosterone, whose blood levels in males are about 20 nM (MW 288.42; 5.71 ± 0.84 ng/ml)^42^. On the other hand, blood triglyceride is lipolytic to fatty acids before passing through the microvessels to be up-taken by parietal cells, and blood fatty acid levels are about 500 µM (474 ± 252.5 µM)^43^. Therefore, isolated gastric gland epithelium was incubated in DMEM with or without testosterone (20 nM) or lauric acids (500 µM) at 37 °C for 1 h (Fig. 4d). Phospholipid levels reflect the amount of plasma membrane, so E2 levels in the gastric gland epithelium including culture medium were normalized by their phospholipid levels. Normalized E2 levels were then compared to those incubated with 20 nM testosterone but without lauric acid. Both testosterone and lauric acid were required for gastric gland epithelium to produce E2, indicating that parietal cells produce estrogen from testosterone in a fatty acid-dependent manner, even in the absence of the possible indirect effects of fatty acids outside the cells. Therefore, our data collectively conclude that blood triglyceride levels can directly regulate the production of gastric estrogen and the following blood estrogen levels in males, even in the absence of possible indirect effects of fatty acids.

### Stomach of OVX female rats, like of male rats, secretes estrogen in response to the blood triglyceride levels

We then examined the effect of blood triglyceride levels on the blood estrogen levels in females. As blood estrogen levels change in the menstrual cycle, 8-week-old OVX female rats were used to exclude the effect of estrogen from the ovaries.

**Fig. 5:**
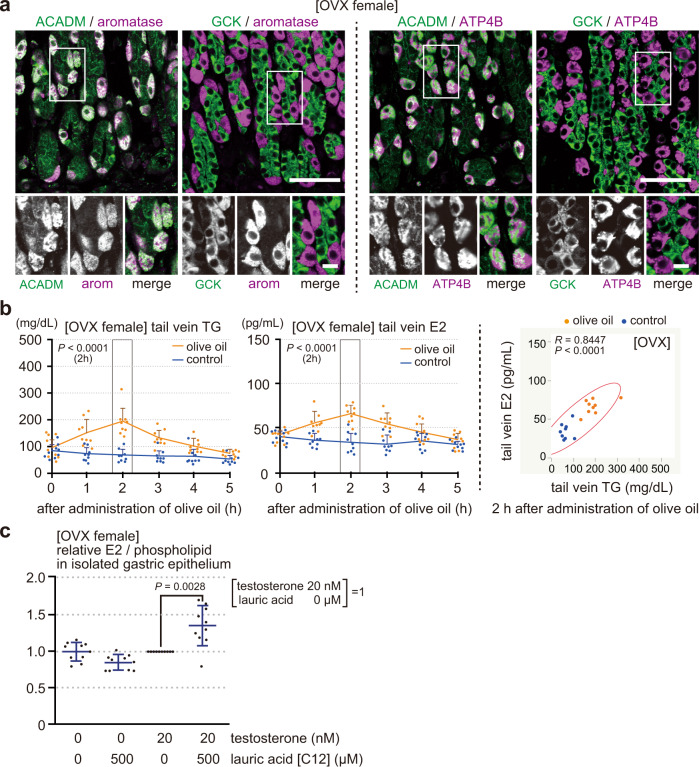
Stomach of OVX female rats, like of male rats, secretes estrogen in response to the blood triglyceride levels. **a** Gastric mucosa from an OVX female rat (8 weeks old, operated 14 days before) was double-stained for ACADM or GCK (green) with aromatase [arom] or ATP4B (magenta). **b** OVX female rats, deprived of food for 4 h, were orally administered olive oil (2.5 mL per kg body weight, *n* = 9) or control water (*n* = 9). TG and E2 levels in the tail venous blood measured before (0) and at 1, 2, 3, 4, and 5 h after the administration (left), and the correlation diagram between the blood TG and E2 levels at 2 h after the administration (*n* = 18, right). **c** Isolated epithelium from OVX female rat (deprived of food for 4 h) was incubated in DMEM with or without testosterone (20 nM) or lauric acid (500 µM) at 37 °C for 1 h. E2 levels, normalized by phospholipid, were compared to those incubated with 20 nM testosterone but without lauric acid (*n* = 10). *n* number of rats. Data were mean ± s.d. **b** left (2 h) and **c** *P* values determined by two-sided Student’s *t*-test. **b** right, *R* and *P* values determined by Pearson’s product-moment correlation with 95% density ellipse. Bars: (main panels) 50 µm; (insets) 10 µm.

First, we examined the energy source of parietal cells in OVX female rats (Fig. 5a). Parietal cells of OVX female rats exclusively expressed aromatase, dominantly expressed ACADM, but not GCK, like in males (Fig. 1c, d). Thus, parietal cells of OVX female rat was also confirmed to produce estrogen using fatty acids, but not glucose, as an energy source.

Next, we examined the effect of blood triglyceride levels on the blood E2 levels in OVX females. Triglyceride (olive oil, 2.5 mL per kg body weight) or control water was orally administered to OVX female rats (deprived of food for 4 h), and their tail venous triglyceride and E2 levels were measured before (0 h) and at 1, 2, 3, 4, and 5 h after the administration (Fig. 5b). Like in male rats (Fig. 2a, b), blood triglyceride and E2 levels increased, peaked at 2 h post-administration, and then declined to the basal levels. There was a positive correlation between them at 2 h after the administration. Thus, blood E2 levels increase as blood triglyceride levels rise in OVX female rats.

Finally, we examined whether fatty acids could directly regulate the production of gastric estrogen in OVX females. OVX female gastric gland epithelium was incubated with or without testosterone (20 nM) or lauric acid (500 µM) at 37 °C for 1 h (Fig. 5c). Normalized E2 levels by phospholipids were compared to those incubated with 20 nM testosterone but without lauric acid. Both testosterone and lauric acid were required for the epithelium to produce E2. Thus, parietal cells from OVX female rats can produce estrogen from testosterone in a fatty acid-dependent manner, even in the absence of the possible indirect effects of fatty acids outside the cells. Therefore, our data conclude that blood triglyceride levels directly regulate the production of gastric estrogen and blood estrogen levels in OVX females, as in males.

## Discussion

Mammals receive body energy status to maintain body energy homeostasis^1^. Ghrelin from the stomach, leptin from adipose tissues, and vagus afferent nerves from the intestines and the liver report the levels of fasting, body fat, and ingested lipids, respectively. They play a part in activation (ghrelin) or inhibition (leptin and vagus afferent nerves) of the NPY neurons^3,4,6^. Activated hypothalamic NPY neurons, in turn, increase the body lipid levels by enhancing the feeding behavior, hepatic de novo lipogenesis, and fatty acid release from adipocytes^19,44–46^. On the other hand, estrogen also influences the lipid homeostasis by inhibiting hypothalamic NPY neurons and de novo lipogenesis in the liver and adipose tissues^14,15,18^, by increasing leptin secretion, WAT mass, and adipogenesis in adipose tissues^17^, and by increasing the uptake and the following β-oxidation of fatty acids in the heart and skeletal muscles^20^. Little is known, however, about the hormones or nerve fibers that inform the blood lipid levels, and about the roles of estrogen in terms of lipid homeostasis.

In this study, we showed that blood triglyceride levels directly regulate the production of gastric estrogen and the following blood estrogen levels, using male and OVX female rats. Parietal cells in the stomach exclusively produce estrogen among the tissues upstream of the portal vein. Production of estrogen requires energy which parietal cells generate using triglyceride. Blood estrogen levels increase as blood triglyceride levels are rised by the oral or intravenous administration of triglyceride. Blood triglyceride-dependent increase of blood estrogen levels is canceled in GX rats. Amounts of estrogen in stomach tissues and isolated gastric gland epithelium depend on the blood triglyceride and medium fatty acid (lauric acid) levels, respectively.

**Fig. 6:**
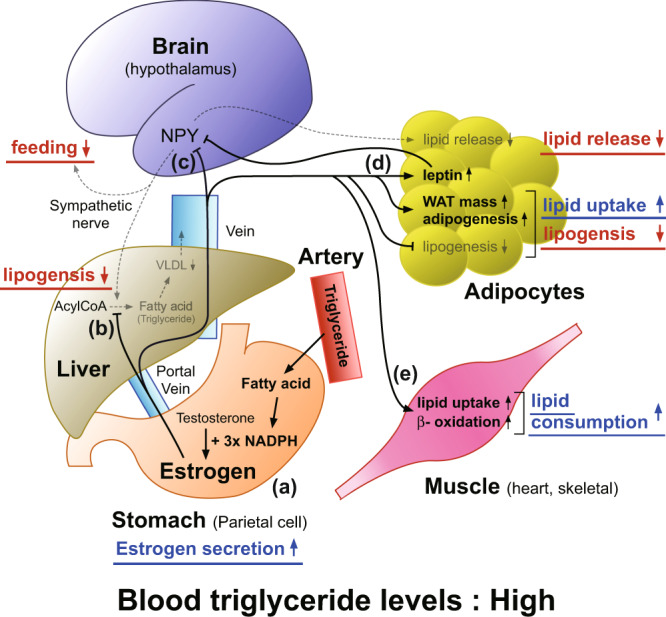
Proposed model for the role of gastric estrogen in maintaining proper blood triglyceride levels. **a** Parietal cells in the stomach produce estrogen using fatty acid (triglyceride) as an energy source (NADPH), and secretion of gastric estrogen increases as blood triglyceride levels rise (this study). **b** Gastric estrogen, secreted into the portal vein, primarily acts on the liver to suppress hepatic de novo lipogenesis^15^. **c** Gastric estrogen inhibits hypothalamic NPY neurons, whose activation enhances feeding behavior, hepatic de novo lipogenesis, and fatty acid release from adipocytes via sympathetic nerves^19, 44–47^. **d** Gastric estrogen acts on adipocytes to suppress their de novo lipogenesis and to increase leptin secretion, white adipose tissue (WAT) mass, and adipogenesis^17^. Leptin, in turn, inhibits hypothalamic NPY neurons^5^. **e** Gastric estrogen increases the lipid uptake and β-oxidation of heart and skeletal muscles^20^. **a**–**e** When blood triglyceride levels are high, secretion of gastric estrogen increases. Increased estrogen inhibits feeding behavior, de novo lipogenesis, and lipid release from adipose tissues while promoting lipid uptake by WAT and lipid consumption by muscles. As a result, blood triglyceride levels are lowered. Conversely, when blood triglyceride levels are low, secretions of gastric estrogen are decreased to supply more lipid to the circulation.

Among the organs upstream of the portal vein, the stomach and the pancreas do not take up nutrients. Therefore, these organs are suitable for sensing the levels of “circulating” nutrients to notify the liver. Indeed, the pancreas senses the blood glucose levels and secretes insulin or glucagon to keep the proper blood glucose levels primarily by acting on the liver. We, therefore, propose a model that the stomach, a digestive organ, acts as a blood triglyceride level sensor to keep proper blood triglyceride levels by secreting estrogen in response to the blood triglyceride levels (Fig. 6). When blood triglyceride levels rise, parietal cells increase the secretion of estrogen into the portal vein, which, in turn, inhibits the *de novo* lipogenesis and feeding behavior, enhances the uptake of lipid by adipose tissues, and the consumption of fatty acids in muscles^17,20^. Estrogen also decreases the lipid release from adipose tissues through the inhibition of NPY neurons^47^. As a result, blood triglyceride levels decrease. Conversely, when blood triglyceride levels lower, parietal cells decrease the secretion of estrogen to supply more lipid to the circulation.

Our model is consistent with the previous reports on the circadian rhythms of blood estrogen and hepatic lipogenesis. At night, secretion of gastric estrogen and blood estrogen levels increase^48^. On the other hand, hepatic lipogenesis decreases at night, which is directly regulated by the circadian feeding (blood triglyceride level) rhythms and rises when blood triglyceride levels are low^49^. As rats actively eat at night, raised blood triglyceride levels would increase the blood estrogen levels, which, in turn, inhibited the hepatic lipogenesis at night.

We showed that the stomach controls the blood estrogen levels in response to the blood triglyceride levels. On the other hand, adipose tissues also secrete estrogen and dominantly use fatty acids like parietal cells (Supplementary Fig. 1c). However, parietal cells contain few, if any, lipid droplets^50^, whereas adipocytes are filled with triglycerides. Thus, intracellular triglyceride levels are more susceptible to the extracellular (blood) triglyceride levels in parietal cells than in adipocytes. We, therefore, think that parietal cells, rather than adipocytes, are suitable for functioning as a sensor for the blood triglyceride levels.

Gastric parietal cells of female rats secrete estrogen and increase the estrogen levels in the portal vein^12^, and we confirmed the secretion of estrogen from female parietal cells in response to the blood triglyceride levels in vivo and in vitro using OVX female rats (Fig. 5). Indeed, blood triglyceride levels are lower in women than in men, but rise after menopause which increases their cardiovascular risks^51^. Thus, estrogen would play important roles in lowering blood triglyceride levels in females, too. However, estrogen and progesterone, a precursor for gastric estrogen, are mainly secreted from the ovaries and their blood levels change dramatically throughout the menstrual cycle. Therefore, further investigations considering the menstruous cycle are required for the precise understanding of the function and regulation of gastric estrogen in females.

We showed that the blood triglyceride levels can “directly” control the secretion of gastric estrogen and the following blood estrogen levels. However, our data do not exclude the presence of “indirect” effects of fatty acids on the secretion of gastric estrogen. Fatty acids activate several signaling pathways, such as fatty acid receptors (e.g., GPR120 and CD36)^33,34^, gastrointestinal hormones (e.g., CCK, GLP1, GIP, PYY, and Ghrelin)^3–5^, and nerves (e.g., vagal afferents)^2,6^. Several kinds of hormones, such as somatomedin, GLP1, and GIP, “indirectly” regulate the secretion of insulin^52^, so the secretion of gastric estrogen might also be “indirectly” regulated by certain kinds of hormones. Indeed, single-cell analysis of parietal cells revealed their higher expression of CCK-B receptor^53^. Therefore, further investigations about the crosstalk between gastric estrogen and other hormones are important to understand the comprehensive hormonal network, which orchestrates the maintenance of energy homeostasis, especially lipids.

## Materials and methods

### Animals

Wistar rats were purchased from Kiwa Laboratory Animals Co., Ltd. (Wakayama, Japan). Animals were housed in an air-conditioned environment (24 ± 2 °C and 50–60% humidity) with 12 h light/dark cycle (lights on at 8:00) and allowed access to food (MF diet, Oriental Yeast Co. Ltd., Tokyo, Japan) and water ad libitum unless otherwise stated. All experiments were conducted according to the protocol approved by the Wakayama Medical University Animal Care and Use Committee.

### Immunoblotting

Crude extracts of the stomach, duodenum, jejunum, ileum, colon, and pancreas were obtained from 8-week-old male rats. Proteins (10 µg for aromatase, 1 µg for β-actin) were separated by SDS-PAGE, transferred to a polyvinylidene fluoride membrane (Immobilon-P, Millipore), and detected with antibodies against aromatase (MCA2077S, 1:250, BioRad) and β-actin (A5060, 1:500, Sigma-Aldrich). Band intensities were quantified with LuminoGraph I (ATTO) using a standard enhanced chemiluminescence protocol. Processing of the images was performed with ImageJ 1.52 (National Institutes of Health).

### Fluorescence staining

We performed immunostaining of rat tissue sections using a standard protocol. Male or OVX female rats of 8 week old were anesthetized using isoflurane and fixed by perfusion with 4% paraformaldehyde in 0.1 M phosphate buffer (pH 7.2). Tissues were then embedded in paraffin, sectioned at 5 µm thickness using a microtome (model RX-860; Yamato Kohki Industrial), and subjected to heat-induced epitope retrieval treatment (pH 6.0) according to the standard method. After blocking with Block One Histo (Nacalai Tesque), sections were incubated with the following primary antibodies against aromatase (MCA2077S, 1:100, BioRad), ATP4B (MA3-923, 1:4,000, Invitrogen), ACADM (GTX100488, 1:200, GeneTex), GCK (HPA007093, 1:50, Sigma-Aldrich), H-FABP (10676-1-AP, 1:250, Proteintech), I-FABP (MAB22970, 1:100, Abnova), GPR120 (NBP1-00858, 1:1000, Novus), CD36 (ab252923, 1:250, Abcam), GLUT1 (ab115730, 1:250, Abcam), insulin (66198-1-Ig, 1:200, Proteintech), amylase (sc-46657, 1:250, Santa Cruz), and perilipin-1 (651156, 1:5, ProGen). Sections were then incubated with Alexa Fluor-conjugated secondary antibodies from Thermo Fisher. After quenching the autofluorescence using TrueView from Vector Laboratory, samples were observed under an LSM-700 confocal laser-scanning microscope equipped with C-Apochromat 40/1.2W or Pan-Apochromat 20/0.8 lens (Carl Zeiss). Image processing and analyses were performed with ImageJ 1.52 (National Institutes of Health).

### Gastrectomy and ovariectomy

Total gastrectomy (GX) was performed on 7-week-old male rats by anastomosing the duodenum and esophagus from end to end^54^. Ovariectomy (OVX) was performed on 6-week-old female rats by ligation and dissection of the ovaries^55^. Medetomidine hydrochloride (0.15 mg per kg body weight), midazolam (4 mg per kg body weight), and butorphanol tartrate (5 mg per kg body weight) as anesthesia provided the desired levels of sedation, analgesia, amnesia, and skeletal muscle relaxation. Postoperatively, the animals were treated with subcutaneous injection of SOLDEM-3A (Terumo) to prevent dehydration. GX male and OVX female rats were used for the subsequent studies 5 days and 2 weeks after the surgery, respectively.

### Oral administration of olive oil and glucose

At 8 weeks of age, rats were deprived of food for 4 h before the experiment. Olive oil (“Yoshida”, Yoshida Pharmaceutical Company, Tokyo, Japan) or control distilled water was orally administered, and blood samples from the tail vein were collected at 0 (before), and 1, 2, 3, 4, and 5 h after the administration^28^. GX rats showed a weaker increase of blood triglyceride levels than normal rats when administered olive oil, so a smaller amount of olive oil was applied in the normal rats (2.5 mL per kg body weight) compared with the GX rats (5 mL per kg body weight). In some studies, rats were sacrificed 2 h after the administration to obtain stomach tissues. Regarding oral administration of glucose, 8-week-old rats, deprived of food for 18 h, were orally administered glucose (2 g per kg body weight, FUJIFILM) or control distilled water^32^. Blood samples from tail veins were collected at 0 (before) and 0.5, 1, 1.5, 2, 3, 4, and 5 h after the administration. We used the gavage technique in the oral administration studies and excluded the rats whose tail venous triglyceride levels before the administration were higher than 200 mg/dL.

### Intravenous administration of triglyceride

At 8 weeks of age, rats were deprived of food for 4 h before the experiment. Twenty-percent soy oil emulsion for intravenous injection (Intralipos Injection 20%, Otsuka, Tokyo, Japan) or control saline was injected to the proximal tail vein (2 mL per kg body weight), and blood samples were collected from the distal tail vein at 0 (before), and 0.1, 0.5, 1, 1.5, 2, 3, 4, and 5 h after the injection. We excluded the rats whose tail venous triglyceride levels before the administration were higher than 200 mg/dL.

### Isolation of gastric gland epithelium

Gastric gland epithelium was isolated according to Mahe’s protocols with slight modifications^56^. At 8 weeks of age, a rat was deprived of food for 4 h before the experiment. The stomach, removed from the anesthetized rat with isoflurane, was opened along the greater curvature and was washed with ice-cold PBS. After removing the serosal muscle, fundic region was cut into <5 mm pieces and was shaken in chelating buffer (5 mM EDTA in PBS) for 2 h on ice. The supernatant was changed with dissociation buffer (54.9 mM d-sorbitol, 43.4 mM sucrose in PBS), and the tube was shaken vigorously for 2 min to dissociate epithelium from the mucosa. After centrifugation at 150 × *g* for 10 min at 4 °C, the pellet was dissolved in DMEM. After filtration with 100 µm cell strainer, the epithelium-rich fractions (Fig. 4b) were incubated in DMEM with or without testosterone (20 nM) or lauric acid (500 µM) at 37 °C for 1 h. E2 and phospholipid levels in the epithelium with culture medium were measured, and E2 levels were normalized by phospholipid levels.

### Measurement of triglyceride, cholesterol, glucose, estrogen, and phospholipid concentrations

Plasma triglyceride, cholesterol, and blood glucose concentrations were measured using a LabAssay Triglyceride (FUJIFILM), a LabAssay Cholesterol (FUJIFILM), and a FreeStyle Libre (Abbott Japan) according to the manufacturer’s instructions, respectively. Total estrogen (E2) concentrations in the lipid fractions of plasma, stomach tissues, and gastric gland epithelium were measured using an Estradiol ELISA kit (Cayman Chemical Company) according to the manufacturer’s instruction. Phospholipid concentrations in the lipid fractions of gastric gland epithelium were measured using a LabAssay Phospholipid (FUJIFILM). The lipid fractions of plasma were prepared as follows. Plasma were mixed with x 4 volume of methanol and incubated at room temperature for 10 min. After centrifugation at 2000 × *g* for 10 min, the supernatants were transferred to new tubes, dried, and reconstituted in the assay buffer. The lipid fractions of stomach tissues and gastric gland epithelium were prepared according to the Bligh & Dyer method^57^. Stomach tissues homogenized in PBS or gastric gland epithelium in culture medium were mixed with x 3.75 volume of chloroform/methanol (1: 2), incubated at room temperature for 10 min, mixed with x 1.25 volume of chloroform, and with x 1.25 volume of distilled water. After centrifugation at 2000 × *g* for 10 min, the lower phase was transferred to a new tube, dried, and reconstituted in the assay buffer.

### Statistics and reproducibility

Data were mean ± s.d. *P* values of different two groups were determined by a two-sided Student’s *t*-test. *R* and *P* values between triglyceride (tail vein) and E2 (tail vein or stomach) levels were determined by Pearson’s product-moment correlation with 95% density ellipse. Statistical analyses were performed using JMP Pro ver. 14 (SAS Institute Japan). *P* values <0.05 were considered to be significant. *R* values >0.5 were considered to be positively correlated. Every experiments was performed multiple times with essentially the same results.

### Reporting summary

Further information on research design is available in the Nature Research Reporting Summary linked to this article.

## Supplementary information

Peer Review File

Supplementary Information

Description of Additional Supplementary Files

Supplementary Data 1

Reporting Summary

## Data Availability

The source data for the figures are available in Supplementary Data 1. Uncropped blots are shown in Supplementary Fig. 2. All other data that support the findings of this study are available from the corresponding author on reasonable request.
