# Supplementary material for: Stomach secretes estrogen in response to the blood triglyceride levels
Source: Commun Biol. 2021 Dec 7;4:1364. doi: 10.1038/s42003-021-02901-9 (PMC8651635; doi:10.1038/s42003-021-02901-9)
Supplement: Supplementary file 2 — Supplementary Information [file 42003_2021_2901_MOESM2_ESM.pdf]

Stomach secretes estrogen in response to the blood triglyceride levels

Takao Ito[1,+], Yuta Yamamoto[1,+], Naoko Yamagishi[1,+], and Yoshimitsu Kanai[1,+,*]
[+] These authors contributed equally

[1] Cell Biology and Anatomy, Graduate School of Medicine, Wakayama Medical University, Wakayama, Japan

* Corresponding Author: ykanai@wakayama-med.ac.jp

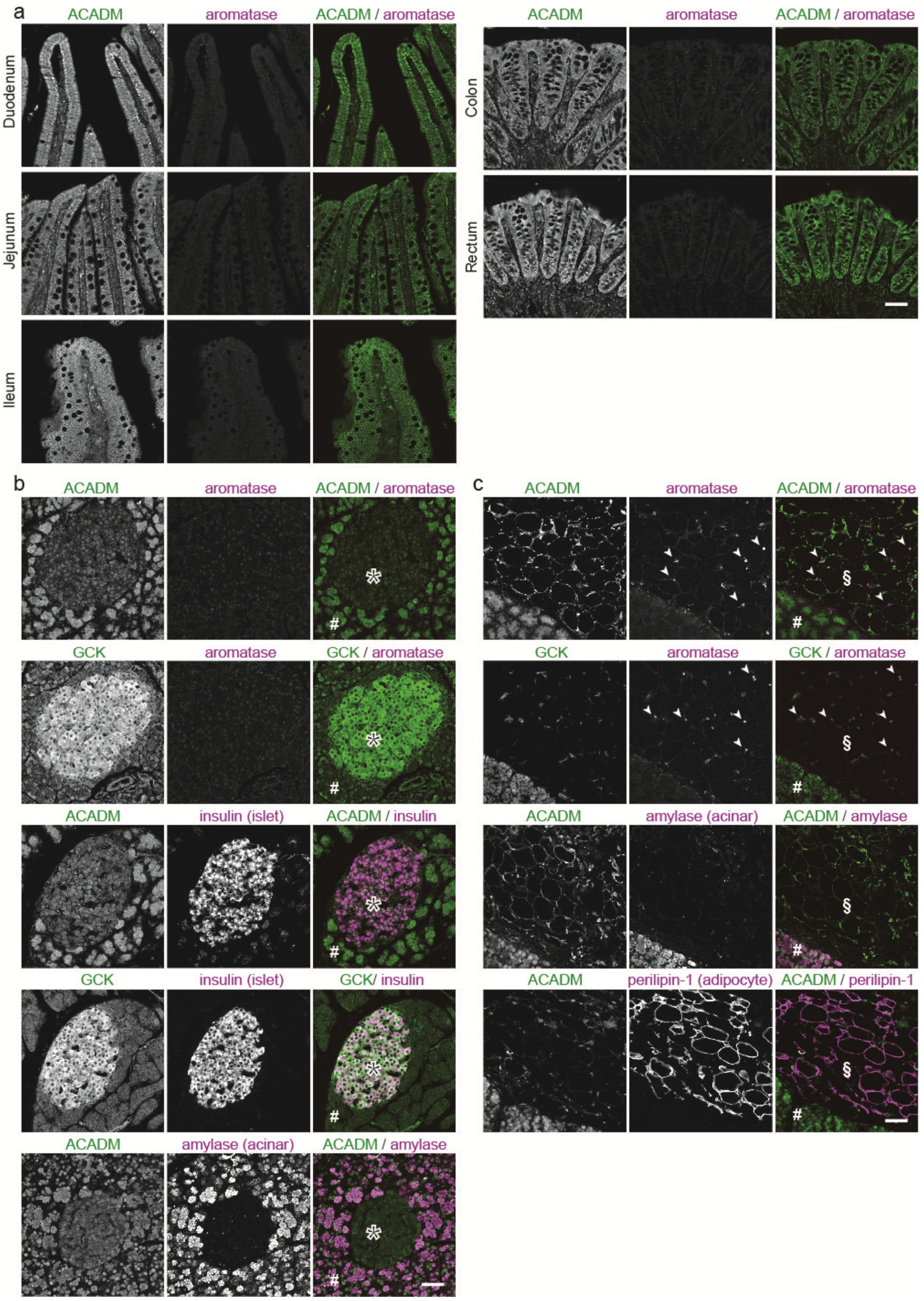

Supplementary Figure 1. Intestines and pancreas do not express aromatase, except for the attached adipocytes. a, Rat duodenums, jejunums, ileums, colons and rectums were double stained for ACADM (green) with aromatase (magenta). **b**, Central regions of rat pancreas were double stained for ACADM or GCK (green) with aromatase, insulin (islet marker; "*") or amylase (acinar cell marker; "#") (magenta). **c**, Peripheral regions of rat pancreas were double stained for ACADM or GCK (green) with aromatase, amylase (acinar cell marker; "#") or perilipin-1 (adipocyte marker; "§") (magenta). Arrowheads indicate the strong expression of aromatase in adipocytes. Bars: 50 μm.

Aromatase

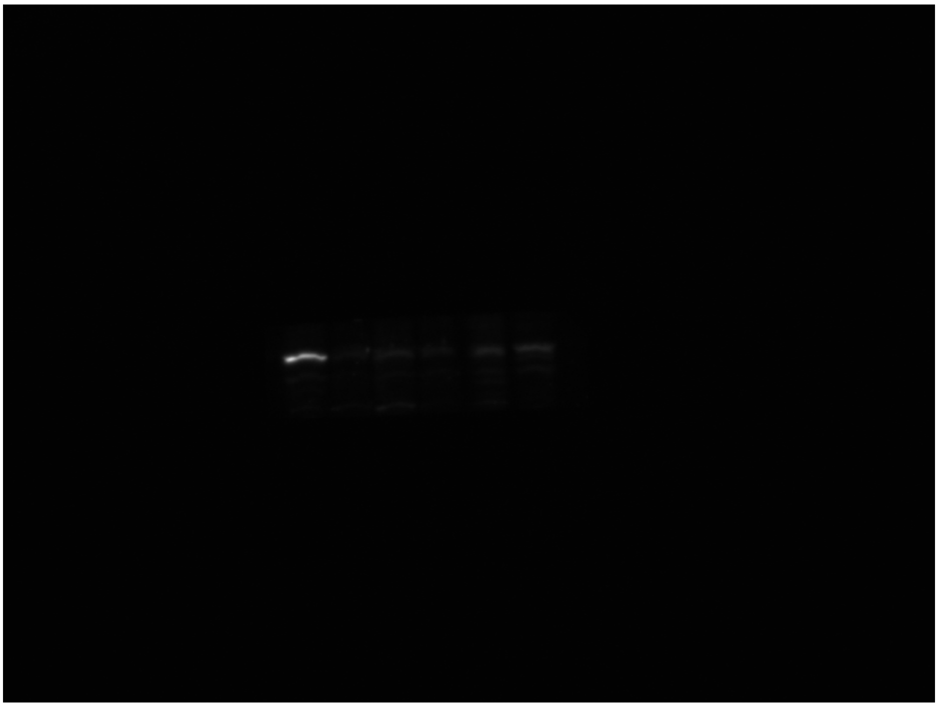

- 55 kDa
- 35 kDa

β-actin

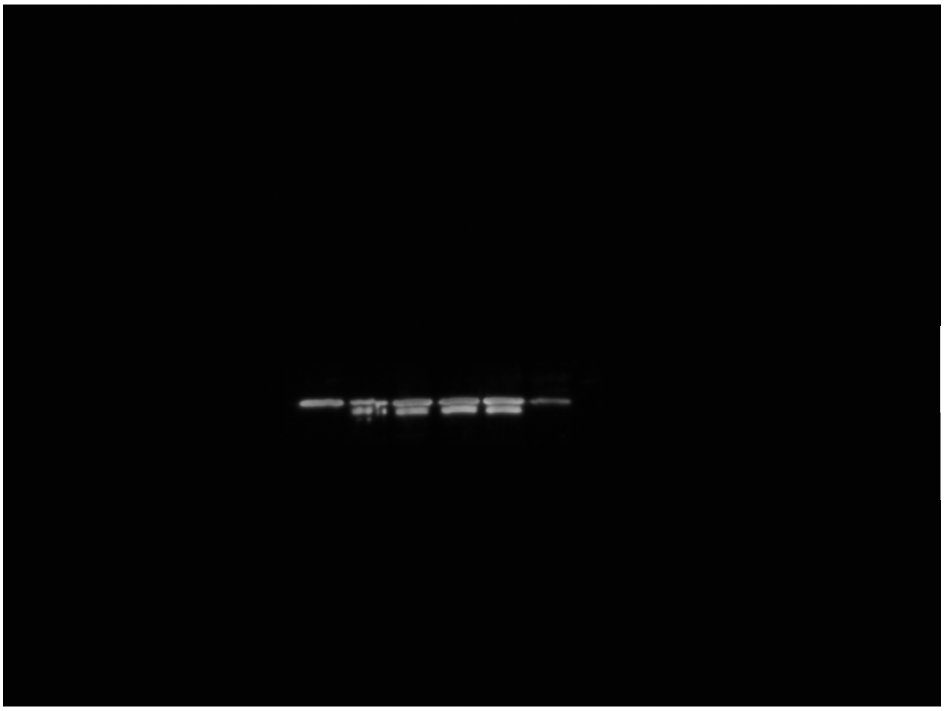

- 55 kDa
- 35 kDa

Supplementary Figure 2. Raw western blots data.
Uncropped and unedited images of western blots in Figure lb.
