# Supplementary material for: Stomach secretes estrogen in response to the blood triglyceride levels
Source: Commun Biol. 2021 Dec 7;4:1364. doi: 10.1038/s42003-021-02901-9 (PMC8651635; doi:10.1038/s42003-021-02901-9)
Supplement: Supplementary file 3 — Description of Additional Supplementary Files [file 42003_2021_2901_MOESM3_ESM.pdf]

Description of Additional Supplementary Files

File name: Supplementary Data 1.
Description: Raw data for figures 2-5.

Sheet 1 (Normal rat) Raw data for figures 2a, b and 3a: TG, E2 and cholesterol levels of tail venous blood (0 [before], 1, 2, 3, 4 and 5 h after administration), and E2 levels in stomach tissues (2 h after administration) of normal male rats administered olive oil (N-oil-[number]) or control water (N-DW-[number]).

Sheet 2 (GX rat) Raw data for figures 3b, c: TG and E2 levels of tail venous blood of GX male rats 0 (before), 1, 2, 3, 4 and 5 h after administration of olive oil (GX-oil-[number]) or control water (GX-DW-[number]).

Sheet 3 (Glu) Raw data for figure 2c: Glucose, E2 and TG levels of tail venous blood of normal male rats 0 (before), 0.5, 1, 1.5, 2, 3, 4 and 5 h after administration of glucose (N-Glu-[number]) or control water (N-DW-[number]).

Sheet 4 (i.v. TG) Raw data for figure 4a: TG and E2 levels of tail venous blood of normal male rats 0 (before), 0.1, 0.5, 1, 1.5, 2, 3, 4 and 5 h after intravenous injection of soy oil (N-oil-[number]) or control saline (N-saline-[number]).

Sheet 5 (Male GM) Raw data for figure 4d: TG and phospholipid levels of isolated gastric mucosa from male rats, incubated in DMEM with or without testosterone (T, 20 nM) or C12 lauric acid (C, 500 μM).

Sheet 6 (OVX rat) Raw data for figure 5b: TG and E2 levels of tail venous blood of OVX female rats 0 (before), 1, 2, 3, 4 and 5 h after administration of olive oil (OVX-oil-[number]) or control water (OVX-DW-[number]).

Sheet 7(OVX GM) Raw data for figure 5c: TG and phospholipid levels of isolated gastric mucosa from OVX female rats, incubated in DMEM with or without testosterone (T, 20 nM) or C12 lauric acid (C, 500 μM).
