# Supplementary material for: Stomach secretes estrogen in response to the blood triglyceride levels
Source: Commun Biol. 2021 Dec 7;4:1364. doi: 10.1038/s42003-021-02901-9 (PMC8651635; doi:10.1038/s42003-021-02901-9)
Supplement: Supplementary file 5 — Reporting Summary [file 42003_2021_2901_MOESM5_ESM.pdf]

nature research

Corresponding author(s): Yoshimitsu Kanai

Last updated by author(s): Nov 8, 2021

Reporting Summary

Nature Research wishes to improve the reproducibility of the work that we publish. This form provides structure for consistency and transparency in reporting. For further information on Nature Research policies, see our Editorial Policies and the Editorial Policy Checklist.

Statistics

For all statistical analyses, confirm that the following items are present in the figure legend, table legend, main text, or Methods section.

n/a	Confirmed	
☐	☒	The exact sample size (*n*) for each experimental group/condition, given as a discrete number and unit of measurement
☐	☒	A statement on whether measurements were taken from distinct samples or whether the same sample was measured repeatedly
☐	☒	The statistical test(s) used AND whether they are one- or two-sided<br>*Only common tests should be described solely by name; describe more complex techniques in the Methods section.*
☒	☐	A description of all covariates tested
☒	☐	A description of any assumptions or corrections, such as tests of normality and adjustment for multiple comparisons
☐	☒	A full description of the statistical parameters including central tendency (e.g. means) or other basic estimates (e.g. regression coefficient) AND variation (e.g. standard deviation) or associated estimates of uncertainty (e.g. confidence intervals)
☒	☐	For null hypothesis testing, the test statistic (e.g. F, t, r) with confidence intervals, effect sizes, degrees of freedom and P value noted<br>*Give P values as exact values whenever suitable.*
☒	☐	For Bayesian analysis, information on the choice of priors and Markov chain Monte Carlo settings
☒	☐	For hierarchical and complex designs, identification of the appropriate level for tests and full reporting of outcomes
☐	☒	Estimates of effect sizes (e.g. Cohen's d, Pearson's r), indicating how they were calculated

Our web collection on statistics for biologists contains articles on many of the points above.

Software and code

Policy information about availability of computer code

Data collection	No custom osoftware or code was used to collect data.
Data analysis	ImageJ (v.1.53) for image analysis, JMP Pro (v.14) for statistical analysis

For manuscripts utilizing custom algorithms or software that are central to the research but not yet described in published literature, software must be made available to editors and reviewers. We strongly encourage code deposition in a community repository (e.g. GitHub). See the Nature Research guidelines for submitting code & software for further information.

Data

Policy information about availability of data

All manuscripts must include a data availability statement. This statement should provide the following information, where applicable:

- Accession codes, unique identifiers, or web links for publicly available datasets
- A list of figures that have associated raw data
- A description of any restrictions on data availability

All data supporting the findings of this study are available within this article and in the Supplementary Information or from the corresponding author upon reasonable request.

Field-specific reporting

Please select the one below that is the best fit for your research. If you are not sure, read the appropriate sections before making your selection.

☒ Life sciences ☐ Behavioural & social sciences ☐ Ecological, evolutionary & environmental sciences

For a reference copy of the document with all sections, see nature.com/documents/nr-reporting-summary-flat.pdf

Life sciences study design

All studies must disclose on these points even when the disclosure is negative.

Sample size	About 10 rats were used for the simple comparison.
Data exclusions	Rats whose tail venous blood triglyceride levels >200 mg/dl before the administration studies were excluded.
Replication	We repeated the administration studies 5 or more times (TG/glucose: ~2 rats + control: ~2 rats / experiment) and collected them, and the immunoblot and immunofluorescence studies at least 3 times.
Randomization	Administration of TG/glucose or control was randomly assigned to rats (~2 rats + ~2 rats / experiment)
Blinding	Blood or tissue samples were numbered regardless of the experimental conditions, and their TG or E2 levels were later measured without notifying the experimental conditions.

Reporting for specific materials, systems and methods

We require information from authors about some types of materials, experimental systems and methods used in many studies. Here, indicate whether each material, system or method listed is relevant to your study. If you are not sure if a list item applies to your research, read the appropriate section before selecting a response.

Materials & experimental systems

n/a	Involved in the study	
☐	☒	Antibodies
☒	☐	Eukaryotic cell lines
☒	☐	Palaeontology and archaeology
☐	☒	Animals and other organisms
☒	☐	Human research participants
☒	☐	Clinical data
☒	☐	Dual use research of concern

Methods

n/a	Involved in the study	
☒	☐	ChIP-seq
☒	☐	Flow cytometry
☒	☐	MRI-based neuroimaging

Antibodies

Antibodies used

Antibodies used for immunofluorescence [IF] and western blot [WB]:
Antibody Name (Species, Company, Cat. No.,(Clone No), Dilution for IF or WB)

Aromatase (mouse, Bio Rad, MCA2077S, H4, 1:100 for IF, 1:250 for WB)
beta-actin (rabbit, Sigma-Aldrich, A5060, 1:500 for WB)
H+/K+ ATPase [ATP4B] (mouse, Invitrogen, MA3-923, 2G11, 1:4,000 for IF)
Acyl-CoA dehydrogenase medium chain [ACADM] (rabbit, GeneTex, GTX100488; 1:200 for IF)
Glucokinase [GCK] (rabbit, Sigma-Aldrich, HPA007093, Sigma-Aldrich, 1:50 for IF)
Heart-type FABP [H-FABR, FABP-3] (rabbit, Proteintech, 10676-1-AP, 1:250 for IF)
Intestine-type FABP [I-FABP, FABP-2] (rabbit, Abnova, MAB22970, 1:100 for IF)
G-protein-coupled receptor 120 [GPR120] (rabbit, Novus, NBP1-00858, 1:1,000 for IF)
Cluster of differentiation 36 [CD36] (rabbit, Abcam, ab252923, 1:250 for IF)
Glucose transporter 1 [GLUT1] (rabbit, Abcam, ab115730, 1:250 for IF)
Insulin (mouse, Proteintech, 66198-1-Ig, 1:200 for IF)
Amylase (mouse, Santa Cruz, sc-46657, 1:250 for IF)
Perilipin-1 (mouse, ProGen, 651156, 1:5 for IF)

Validation

Previously published or manufacturer-validated antibodies were used for this study. The manufacturers' websites contain the validation data for these antibodies. We tested all antibodies on PFA-fixed rat tissue samples.

Animals and other organisms

Policy information about studies involving animals; ARRIVE guidelines recommended for reporting animal research

Laboratory animals	Male Wistar rats (6-7 week-old) were purchased from Kiwa Laboratory Animals Co., Ltd. (Wakayama, Japan).
Wild animals	We did not use wild animals.
Field-collected samples	We did not use field collected samples.
Ethics oversight	All rat experiments were conducted according to the protocol approved by the Wakayama Medical University Animal Care and Use Committee.

Note that full information on the approval of the study protocol must also be provided in the manuscript.
