# Supplementary material for: Stomach secretes estrogen in response to the blood triglyceride levels
Source: Commun Biol. 2021 Dec 7;4:1364. doi: 10.1038/s42003-021-02901-9 (PMC8651635; doi:10.1038/s42003-021-02901-9)
Supplement: Supplementary file 1 — Peer Review File [file 42003_2021_2901_MOESM1_ESM.pdf]

nature portfolio

Peer Review File

Stomach secretes estrogen in response to the blood triglyceride levels.

Open Access This file is licensed under a Creative Commons Attribution 4.0 International License, which permits use, sharing, adaptation, distribution and reproduction in any medium or format, as long as you give appropriate credit to the original author(s) and the source, provide a link to the Creative Commons license, and indicate if changes were made. In the cases where the authors are anonymous, such as is the case for the reports of anonymous peer reviewers, author attribution should be to 'Anonymous Referee' followed by a clear attribution to the source work. The images or other third party material in this file are included in the article's Creative Commons license, unless indicated otherwise in a credit line to the material. If material is not included in the article's Creative Commons license and your intended use is not permitted by statutory regulation or exceeds the permitted use, you will need to obtain permission directly from the copyright holder. To view a copy of this license, visit http://creativecommons.org/licenses/by/4.0/.

Reviewers' comments:

Reviewer #1 (Remarks to the Author):

The MS, "stomach secretes estrogen in response to blood triglyceride levels in males", by Ito et al shows that parietal cells of the stomach are very sensitive to blood cholesterol concentrations and they secrete estrogen while blood cholesterol increase and vice versa. They employed immunocytochemical and other techniques to obtain main results of the study. They also administered olive oil at 2.5 mg/l to increase blood TG levels and consequently to demonstrate estrogen secretion from parietal cells of the stomach.
The study is very straight forward; however, I have several questions regarding a few points that need to be addressed.
-Did the investigators used the gavage technique for olive oil administration or the oil was added into the feeder bottles? If the animals were given orally, there might be the activation of fat taste bud sensors in the tongue, leading the information to the brain and then to the stomach via vagus nerve. Recently, there is an upsurge of information on fat taste sensing via tongue CD36 and GPR120 (Ozdener et al., Gastroenterology, 2014; Besnard et al., Physiological Re. 2016).
-Secondly, if oil is given via gavage technique, the fatty acids like oleic acid (and others) can directly activate parietal cells as stomach cells do express CD36 (Chen et al, 2001, Am J Clin Endocrinol Meatb). Besides, stomach cells also express GPR120 and its expression is regulated by a high-fat diet (Widmayer et al., 2015, Front Physiol). Hence, oil administration might activate these lipid sensors, if yes, how, estrogen secretion is regulated?
-Furthermore, the dietary fatty acid might directly interact with gastrin or ghrelin secreting cells in the stomach? How will there be an impact on estrogen secretion?
-Besides, the investigators completely ignored the secretion of CCK and PYY from intestinal cells. They could have determined their concentrations in oil administration concomitantly with total cholesterol increase and estrogen secretion.
-How tongue-brain-stomach axis is activated or regulated as fat eating behavior immediately regulated by anticipatory mechanisms by gustatory nerves like IV and VII?

Reviewer #2 (Remarks to the Author):

In the manuscript "Stomach secretes estrogen in response to blood triglyceride levels in males", the authors present that gastric parietal cells produce estrogen and mainly use fatty acids as an energy source. The estrogen level in the blood of male rats is positively correlated with the triglyceride level. In gastrectomized rats, increases of blood estrogen levels are vanished, but the levels of serum triglyceride rise.
While the data presented is interesting, this study is too preliminary. There are some concerns need to be determined.

1. Authors stated that parietal cells produced estrogen in response to blood triglyceride levels, based on correlation analysis. The serum triglyceride of gastrectomized rats peaked at 2 hrs post administration of olive oil (~200mg/ml), which was approximately 1/3 lower than that of normal rats and indicated that gastric absorption of fatty acid was decreased. It is impossible to distinguish whether the increase in blood estrogen is caused by the gastric absorption of fatty acids or by the increase in blood triglycerides.
2. The age of rats used in this study was not mentioned throughout the manuscript. And I wonder if estrogen levels in male rats change with age, just like in female rats.
3. The ovaries are the major source of estrogen in female rats. Do gastric parietal cells of normal female rats also produce estrogen? Will the secretion of gastric estrogen increase in ovariectomized female rats?
4. The blood cholesterol level in male rats treated with olive oil?
5. The major fatty acids in olive oil are oleic acid, linoleic acid and palmitic acid, which are long-chain fatty acid. What is the effect of short-chain fatty acids on blood estrogen levels?
6. The expression of glucose transporter GLUT1 and fatty acid transporters FABPs should be determined.
7. In the olive oil treatment experiment, whether the rats fasted or not?

8. Acinar cells and attached adipocytes should also be co-stained by cell markers, not just based on location and shape of different cell types.

Reviewers' comments:

Reviewer #1 (Remarks to the Author):

The MS, “stomach secretes estrogen in response to blood triglyceride levels in males”, by Ito et al shows that parietal cells of the stomach are very sensitive to blood cholesterol concentrations and they secrete estrogen while blood cholesterol increase and vice versa. They employed immunocytochemical and other techniques to obtain main results of the study. They also administered olive oil at 2.5 mg/l to increase blood TG levels and consequently to demonstrate estrogen secretion from parietal cells of the stomach.
The study is very straight forward; however, I have several questions regarding a few points that need to be addressed.
-Did the investigators used the gavage technique for olive oil administration or the oil was added into the feeder bottles? If the animals were given orally, there might be the activation of fat taste bud sensors in the tongue, leading the information to the brain and then to the stomach via vagus nerve. Recently, there is an upsurge of information on fat taste sensing via tongue CD36 and GPR120 (Ozdener et al., Gastroenterology, 2014; Besnard et al., Physiological Re. 2016).
-Secondly, if oil is given via gavage technique, the fatty acids like oleic acid (and others) can directly activate parietal cells as stomach cells do express CD36 (Chen et al, 2001, Am J Clin Endocrinol Meatb). Besides, stomach cells also express GPR120 and its expression is regulated by a high-fat diet (Widmayer et al., 2015, Front Physiol). Hence, oil administration might activate these lipid sensors, if yes, how, estrogen secretion is regulated?
-Furthermore, the dietary fatty acid might directly interact with gastrin or ghrelin secreting cells in the stomach? How will there be an impact on estrogen secretion?
-Besides, the investigators completely ignored the secretion of CCK and PYY from intestinal cells. They could have determined their concentrations in oil administration concomitantly with total cholesterol increase and estrogen secretion.
-How tongue-brain-stomach axis is activated or regulated as fat eating behavior immediately regulated by anticipatory mechanisms by gustatory nerves like IV and VII?

Reviewer #2 (Remarks to the Author):

In the manuscript "Stomach secretes estrogen in response to blood triglyceride levels in males", the authors present that gastric parietal cells produce estrogen and mainly use fatty acids as an energy source. The estrogen level in the blood of male rats is positively correlated with the triglyceride level. In gastrectomized rats, increases of blood estrogen levels are vanished, but the levels of serum triglyceride rise.
While the data presented is interesting, this study is too preliminary. There are some concerns

need to be determined.

1. Authors stated that parietal cells produced estrogen in response to blood triglyceride levels, based on correlation analysis. The serum triglyceride of gastrectomized rats peaked at 2 hrs post administration of olive oil (~200mg/ml), which was approximately 1/3 lower than that of normal rats and indicated that gastric absorption of fatty acid was decreased. It is impossible to distinguish whether the increase in blood estrogen is caused by the gastric absorption of fatty acids or by the increase in blood triglycerides.
2. The age of rats used in this study was not mentioned throughout the manuscript. And I wonder if estrogen levels in male rats change with age, just like in female rats.
3. The ovaries are the major source of estrogen in female rats. Do gastric parietal cells of normal female rats also produce estrogen? Will the secretion of gastric estrogen increase in ovariectomized female rats?
4. The blood cholesterol level in male rats treated with olive oil?
5. The major fatty acids in olive oil are oleic acid, linoleic acid and palmitic acid, which are long-chain fatty acid. What is the effect of short-chain fatty acids on blood estrogen levels?
6. The expression of glucose transporter GLUT1 and fatty acid transporters FABPs should be determined.
7. In the olive oil treatment experiment, whether the rats fasted or not?
8. Acinar cells and attached adipocytes should also be co-stained by cell markers, not just based on location and shape of different cell types.

We thank the reviewers and editors for their informative and important suggestions for our work. According to their suggestions, we performed several experiments and updated the figures, which are included at the end of this document.

The major and minor changes are as follows:

Major changes:
(1) The most important concern of the reviewers and editors is ”Is gastric estrogen secretion regulated “directly” by blood triglyceride (TG) levels, or “indirectly” by hormones or nerves stimulated by ingested fatty acids (FA) in gastrointestinal tract?” To address this question, we performed *in vivo* and *in vitro* studies (Fig. 4). First, we increased the blood TG levels by injecting soy oil emulsion into the tail vein (Fig. 4a). Using this method, we passed the ingestion process via gastrointestinal tract and avoided the stimulation of hormones or nerves via ingested FA there, and confirmed the increase of the blood estrogen levels when blood TG levels were high. However, the intravenously injected TG was lipolyzed to FA in the circulation, which in turn, might stimulate the hormones or nerves in the gastrointestinal tract, even if the amount of FA reached the gastrointestinal tract was far less than those in oral administration studies; injected triglyceride was 2 mL/kg BW of 20 % TG emulsion (0.4 mL/kg BW) and was lipolyzed to FA in the circulation, only a part of which enters the gastrointestinal tract before uptaken by adipose tissues or used by muscles (orally administered TG: 2.5 mL/kg BW, all of which was lipolyzed to FA and passed through the gastrointestinal tract). Therefore, we further performed in vitro studies using isolated gastric gland epithelium to avoid the effects outside the epithelium (Fig. 4b). However, as indicated by the reviewer, FA can stimulate CD36 and GPR120 in the stomach, although they are not expressed in parietal cells (Fig. 4c). To escape from the stimulation of these receptors, we incubated gastric gland epithelium with testosterone and lauric acid (C12 FA), which does not interact with these receptors but can bind to H-FABP in parietal cells (Figs. 1e and 4c). Gastric gland epithelium produced estrogen in a lauric acid dependent manner under the presence of testosterone. Therefore, we believe that gastric parietal cells can produce estrogen in response to the blood TG level, even in the absence of the hormones or nerves stimulated by FA. As for the indirect effects of these hormones or nerves on the secretion of gastric estrogen, we think them to be examined one by one in the future studies.

(2) The reviewer #2 pointed out about the secretion of gastric estrogen in females, and suggested the studies using OVX female rats. To address this point, we performed three key experiments using OVX female rats (Fig. 5). [1] We confirmed the expression of aromatase and the dominant use of FA in parietal cells of OVX female rat (Fig. 5a). [2] Blood estrogen levels increased as blood TG levels rose after the oral administration of olive oil (Fig. 5b). [3] Isolated gastric gland epithelium produced estrogen in a lauric acid dependent manner under the presence of testosterone (Fig. 5c). Therefore, we conclude that gastric parietal cells secrete estrogen and increase blood estrogen levels in response to the blood TG levels in OVX female rats, and removed the

terms “in males” from our study title. As for the effects of menstrual cycles, they should be precisely investigated in the future studies.

Minor changes:
(3) Fig. 1e: We investigated the distribution of H-FABP, GLUT1 and I-FABP in the gastric mucosa.
(4) Fig. 2a: We added the blood cholesterol levels in the oral administration of olive oil study.
(5) Fig. 4c: We investigated the distribution of GPR120 and CD36 in the gastric mucosar.
(6) Fig. 6: We added known functions of estrogen on the adipose tissues and muscles in our model and removed one of the panels showing the roles of gastric estrogen when blood TG levels are low.
(7) Supplementary Fig. 1: We used antibodies against amylase and perilipin1 to identify acinar cells and adipocytes, respectively.
(8) We changed the style of IF data to save space.

We addressed the reviewer’s comments (in blue italics) point by point below.

Reviewers' comments:

Reviewer #1 (Remarks to the Author):

The MS, “stomach secretes estrogen in response to blood triglyceride levels in males”, by Ito et al shows that parietal cells of the stomach are very sensitive to blood cholesterol concentrations and they secrete estrogen while blood cholesterol increase and vice versa. They employed immunocytochemical and other techniques to obtain main results of the study. They also administered olive oil at 2.5 mg/l to increase blood TG levels and consequently to demonstrate estrogen secretion from parietal cells of the stomach.
The study is very straight forward; however, I have several questions regarding a few points that need to be addressed.

We would like to thank this reviewer for the encouraging comments on our work.

-Did the investigators used the gavage technique for olive oil administration or the oil was added into the feeder bottles? If the animals were given orally, there might be the activation of fat taste bud sensors in the tongue, leading the information to the brain and then to the stomach via vagus nerve. Recently, there is an upsurge of information on fat taste sensing via tongue CD36 and GPR120 (Ozdener et al., Gastroenterology, 2014; Besnard et al., Physiological Re. 2016).

In the oral administration studies, a gavage technique was used to inject olive oil (glucose, DW) into the stomach [normal rats] or duodenum [GX rats] using a syringe with a tube. Therefore, we

think that the tongue lipid sensor was not activated in these studies.

-Secondly, if oil is given via gavage technique, the fatty acids like oleic acid (and others) can directly activate parietal cells as stomach cells do express CD36 (Chen et al, 2001, Am J Clin Endocrinol Meatb). Besides, stomach cells also express GPR120 and its expression is regulated by a high-fat diet (Widmayer et al., 2015, Front Physiol). Hence, oil administration might activate these lipid sensors, if yes, how, estrogen secretion is regulated?

We thank the reviewer for this remark. We examined the distribution of CD36 and GPR120 in the gastric mucosa (Fig. 4c). They were not expressed in parietal cells, but were expressed in other types of cells in the gastric mucosa. Thus, CD36 or GPR120 would not "directly" regulate the secretion of estrogen in parietal cells. To exclude the "indirect" effects from gastric CD36 / GPR120 expressing cells on the secretion of gastric estrogen, we performed the following *in vivo* and *in vitro* studies.

To exclude the effects of ingested fatty acids from gastric cavity, we increased the blood triglyceride levels by intravenous injection of triglyceride emulsion (Fig. 4a). Intravenously injected triglyceride emulsion rapidly increased the blood triglyceride levels and returned to the basal levels at 1~1.5 h later. Blood estrogen levels also increased rapidly and returned to the basal levels at 1.5~2 h later. Therefore, we think blood estrogen levels increase as blood triglyceride levels rise, even in the absence of the ingested fatty acids through the gastric tract.

Parietal cells use H-FABP for their uptake and use of fatty acids (Iseki et al., 1991, Fig. 1e). H-FABP binds to the fatty acids of C12 to C20, whereas GPR120 and CD36 to those of C14 or above and C20 or above, respectively (Wang et al., 2019, Hirasawa et al., 2005, Drover et al., 2008). This means that lauric acid (C12 fatty acid) can be used in parietal cells as an energy source, but does not stimulate GPR120 or CD36. We then isolated gastric gland epithelium (Fig. 4b) to incubate with testosterone and lauric acid (C12 fatty acid) to avoid the indirect effect from the CD36 and GPR120 expressing cells (Fig. 4d). As isolated gastric gland epithelium produced estrogen only when both testosterone and lauric acid were present , we concluded that parietal cells can "directly" produce estrogen in response to fatty acids, even in the absence of the "indirect" effects of fatty acids from outside.

In this manuscript, we showed that the increases of blood triglyceride levels "directly" induces the secretion of gastric estrogen. Therefore, we think that the indirect effects of gastric GPR120 and CD36 (and other hormones or neurons) on the secretion of gastric estrogen are out of scope in this study and should be examined in the future studies.

-Furthermore, the dietary fatty acid might directly interact with gastrin or ghrelin secreting cells in the stomach? How will there be an impact on estrogen secretion?

As described above, we showed that blood estrogen levels increase when blood triglyceride levels are increased by intravenous injection of triglyceride emulsion (Fig. 4a). Therefore, blood estrogen levels increase when blood triglyceride levels are risen, even in the absence of dietary fatty acids from gastric cavity which might directly interact with gastrin or ghrelin secreting cells in the stomach.

As for the impacts of gastrin or ghrelin on the secretion of gastric estrogen, we think they are out of scope in this study and should be examined in the future studies.

-Besides, the investigators completely ignored the secretion of CCK and PYY from intestinal cells. They could have determined their concentrations in oil administration concomitantly with total cholesterol increase and estrogen secretion.

As described above, we performed intravenous injection of triglyceride emulsion to “directly” increase blood triglyceride levels, avoiding the effects of intestinal CCK and PYY induced by ingested fatty acids (Fig. 4a). Our *in vitro* studies using isolated gastric gland epithelium completely excluded the effects of intestinal CCK and PYY on the production of gastric estrogen (Fig. 4d). Blood estrogen levels increased as blood triglyceride levels are risen by the intravenous injection of triglyceride emulsion, and production of estrogen from isolated gastric gland epithelium required the presence of fatty acids.

As for the blood cholesterol levels, they increased but not significant, as blood triglyceride levels increased (Fig. 2a).

-How tongue-brain-stomach axis is activated or regulated as fat eating behavior immediately regulated by anticipatory mechanisms by gustatory nerves like IV and VII?

As described above, isolated gastric gland epithelium, which is separated from nerves, produced estrogen from testosterone in a fatty acid dependent manner (Fig. 4d). Therefore, blood estrogen levels increase when blood triglyceride levels are risen, even in the absence of innervated nerves.

We think that the indirect effects of neurons on the secretion of gastric estrogen are out of scope in this study and should be examined in the future studies.

Reviewer #2 (Remarks to the Author):

In the manuscript "Stomach secretes estrogen in response to blood triglyceride levels in males", the authors present that gastric parietal cells produce estrogen and mainly use fatty acids as an energy source. The estrogen level in the blood of male rats is positively correlated with the triglyceride level. In gastrectomized rats, increases of blood estrogen levels are vanished, but the

levels of serum triglyceride rise.
While the data presented is interesting, this study is too preliminary. There are some concerns need to be determined.

We thank this reviewer for suggesting many important points which greatly improved our work.

1. Authors stated that parietal cells produced estrogen in response to blood triglyceride levels, based on correlation analysis. The serum triglyceride of gastrectomized rats peaked at 2 hrs post administration of olive oil (~200mg/ml), which was approximately 1/3 lower than that of normal rats and indicated that gastric absorption of fatty acid was decreased. It is impossible to distinguish whether the increase in blood estrogen is caused by the gastric absorption of fatty acids or by the increase in blood triglycerides.

To exclude the possible gastric absorption of fatty acids, we increased the blood triglyceride levels by intravenous injection of triglyceride emulsion (Fig. 4a). Intravenously injected triglyceride emulsion rapidly increased the blood triglyceride levels and returned to the basal levels at 1~1.5 h later. Blood estrogen levels also increased rapidly and returned to the basal levels at 1.5~2 h later. Therefore, we think blood estrogen levels increase as blood triglyceride levels rise, even in the absence of the ingested fatty acids from the gastric cavity.

2. The age of rats used in this study was not mentioned throughout the manuscript. And I wonder if estrogen levels in male rats change with age, just like in female rats.

We described the ages of rats (8-week old at experiments) in the figure legends and method sections. Gastric aromatase and estrogen levels in male rats are reported to increase with age (examined up to 40 days) (Kobayashi et al., J Endocrinology, 218:117, 2013).

3. The ovaries are the major source of estrogen in female rats. Do gastric parietal cells of normal female rats also produce estrogen? Will the secretion of gastric estrogen increase in ovariectomized female rats?

We performed the immunofluorescence study, oral administration of olive oil study, and *in vitro* study using isolated gastric study on OVX female rats (Fig. 5). Parietal cells from OVX rats also exclusively expressed aromatase and used fatty acids, not glucose, as an energy source (Fig. 5a). Blood estrogen levels increased as blood triglyceride levels rose in OVX female rats (Fig. 5b). Isolated gastric gland epithelium from OVX female rats produced estrogen in the presence of testosterone and lauric acids (C12 fatty acid), which can be used as energy source in parietal cells by H-FABP, but does not stimulate GPR120 or CD36 (Fig. 5c).

4. The blood cholesterol level in male rats treated with olive oil?

Blood cholesterol levels increased but not significant as blood triglyceride levels increased (Fig. 2a).

5. The major fatty acids in olive oil are oleic acid, linoleic acid and palmitic acid, which are long-chain fatty acid. What is the effect of short-chain fatty acids on blood estrogen levels?

We performed the *in vitro* studies using isolated gastric gland epithelium with testosterone and lauric acids (C12 fatty acid), which can be used as energy source in parietal cells by H-FABP, but does not stimulate GPR120 or CD36 (Figs. 4d and 5c). Isolated gastric gland epithelium produce estrogen in the presence of testosterone and lauric acids

6. The expression of glucose transporter GLUT1 and fatty acid transporters FABPs should be determined.

We examined the expression of GLUT1, H-FABP, and I-FABP (Fig. 1e). Gastric parietal cells expressed H-FABP, but not GLUT1 or I-FABP.

7. In the olive oil treatment experiment, whether the rats fasted or not?

Rats were fasted for 4 h before the olive oil treatment. We described the fasting information in the figure legends.

8. Acinar cells and attached adipocytes should also be co-stained by cell markers, not just based on location and shape of different cell types.

We used antibodies against amylase and perilipin-1 to identify acinar cells and adipocytes, respectively (Supplementary Figs. 1b and c).

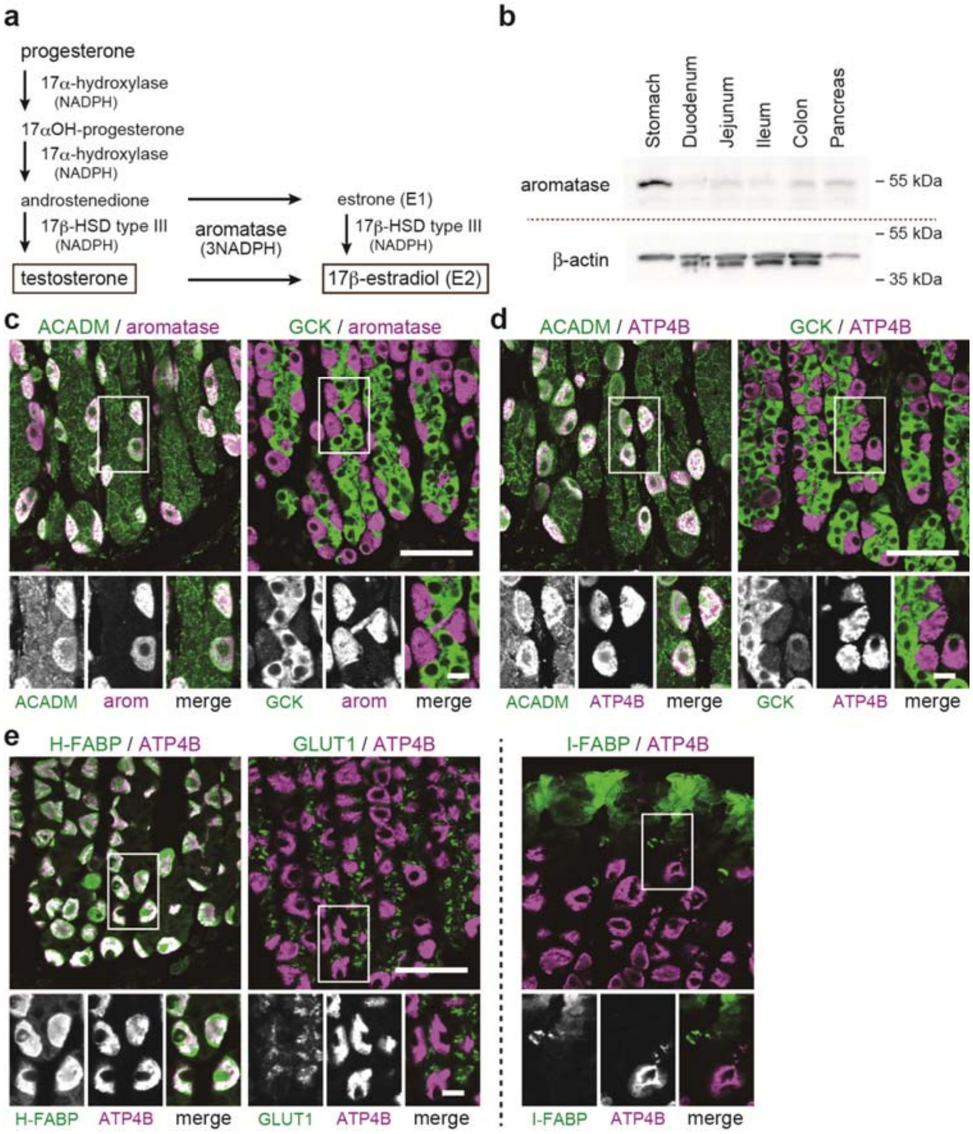

Fig. 1 Estrogen producing gastric parietal cells use fatty acids as an energy source in males. **a**, Scheme of testosterone (progesterone)-17β-estradiol (E2) conversion pathway in rat gastric parietal cells. **b**, Immunoblotting of crude extracts from the stomach, duodenum, jejunum, ileum, colon and pancreas of adult male rats using antibodies against aromatase and control β-actin. **c-e**, Gastric mucosa from male rat was double stained for ACADM (acyl-CoA dehydrogenase medium chain, an enzyme involved in β-oxidation) or GCK (glucokinase, a glycolytic enzyme) (green) with aromatase [arom] (magenta) (**c**), for ACADM or GCK (green) with ATP4B (H^+/K^+ ATPase, a gastric parietal cell marker; magenta) (**d**), and for H-FABP (heart-type fatty acid-binding protein [FABP]), GLUT1 (glucose transporter 1), or I-FABP (intestinal FABP) (green) with ATP4B (magenta) (**e**). Bars: (main panels) 50 µm; (insets) 10 µm.

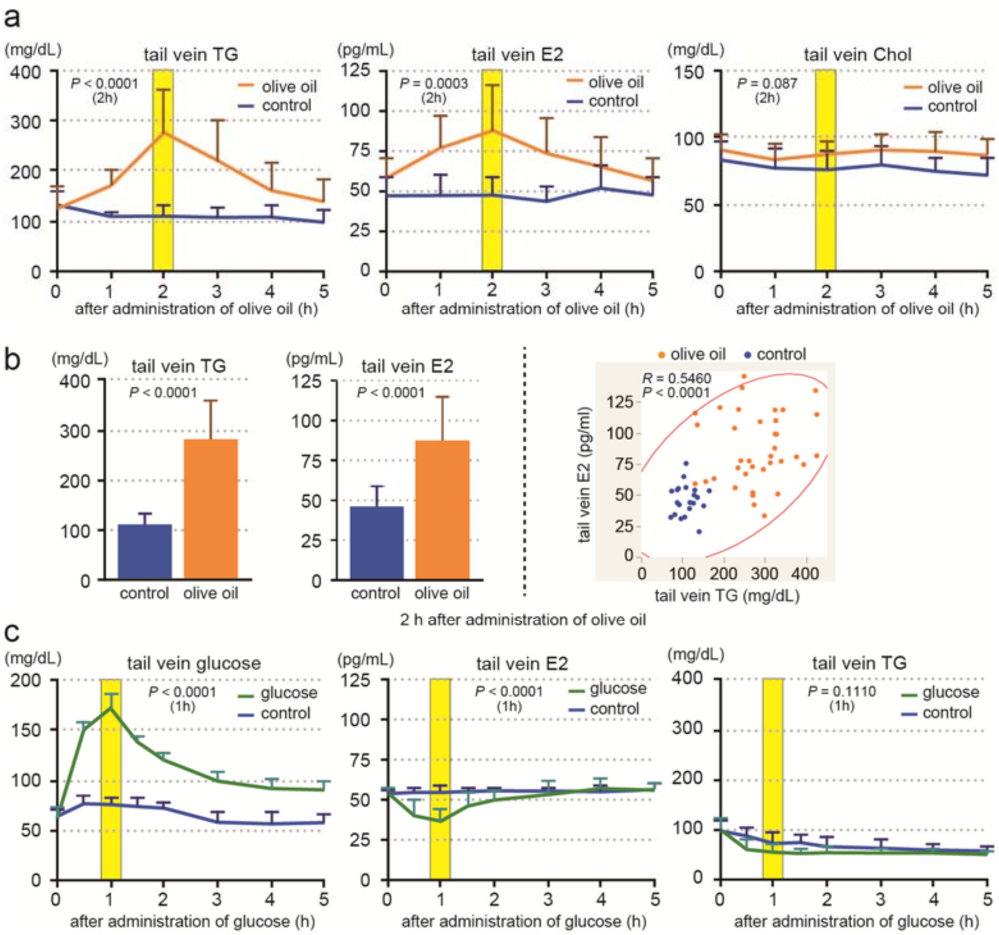

Fig. 2 Blood estrogen levels increase as blood triglyceride levels rise in males. a and **b**, Male rats (8-week-old, deprived of food for 4 h) were orally administered olive oil (2.5 mL per kg body weight) or control water (control) using gavage technique. Triglyceride (TG), E2 and cholesterol (Chol) levels in the tail venous blood were measured before (0) and at 1, 2, 3, 4, 5 h after the administration (olive oil: n = 13, control: n = 8, **a**). TG and E2 levels in the tail venous blood measured at 2 h after the administration (olive oil: n = 37, control: n = 21, **b,** left), and the correlation diagram between the blood TG and E2 levels (n = 58, **b**, right). **c**, Male rats (8-week-old, deprived of food for 18 h) were orally administered glucose (2 g per kg body weight, n = 10) or control water (n = 9). Glucose, E2 and TG in the tail venous blood were measured before (0) and at 0.5, 1, 1.5, 2, 3, 4, 5 h after the administration. n, number of rats. Data are mean ± s.d. P values determined by two-sided Student's t-test at 2 h (**a**, **b**, left) or 1 h (**c**) after the administration. **b**, right, R and P values determined by Pearson's product moment correlation with 95% density ellipse.

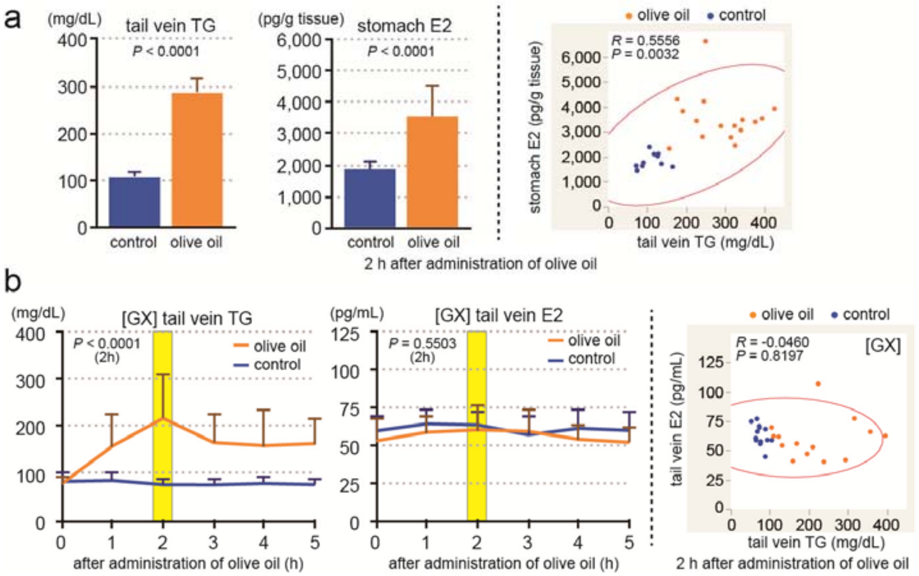

Fig. 3 Stomach secretes estrogen in response to the blood triglyceride levels in males. a, Male rats (8-week-old, deprived of food for 4 h) were orally administered olive oil (2.5 mL per kg body weight, n = 16) or control water (n = 10). TG levels in the tail venous blood and E2 levels in the stomach tissues measured at 2 h after the administration (left), and the correlation diagram between the blood TG and stomach E2 levels (n = 26, right). **b**, GX male rats (8-week-old, operated 5 days before, deprived of food for 4 h) were orally administered olive oil (5 mL per kg body weight, n = 14) or control water (n = 13). TG and E2 levels in the tail venous blood measured before (0) and at 1, 2, 3, 4, 5 h after the administration (left), and the correlation diagram between the blood TG and E2 levels at 2 h after the administration (n = 27, right). n, number of rats. Data are mean ± s.d. **a**, left and **b**, left, P values determined by two-sided Student's t-test at 2 h after the administration. **a**, right and **b**, right, R and P values determined by Pearson's product moment correlation with 95% density ellipse.

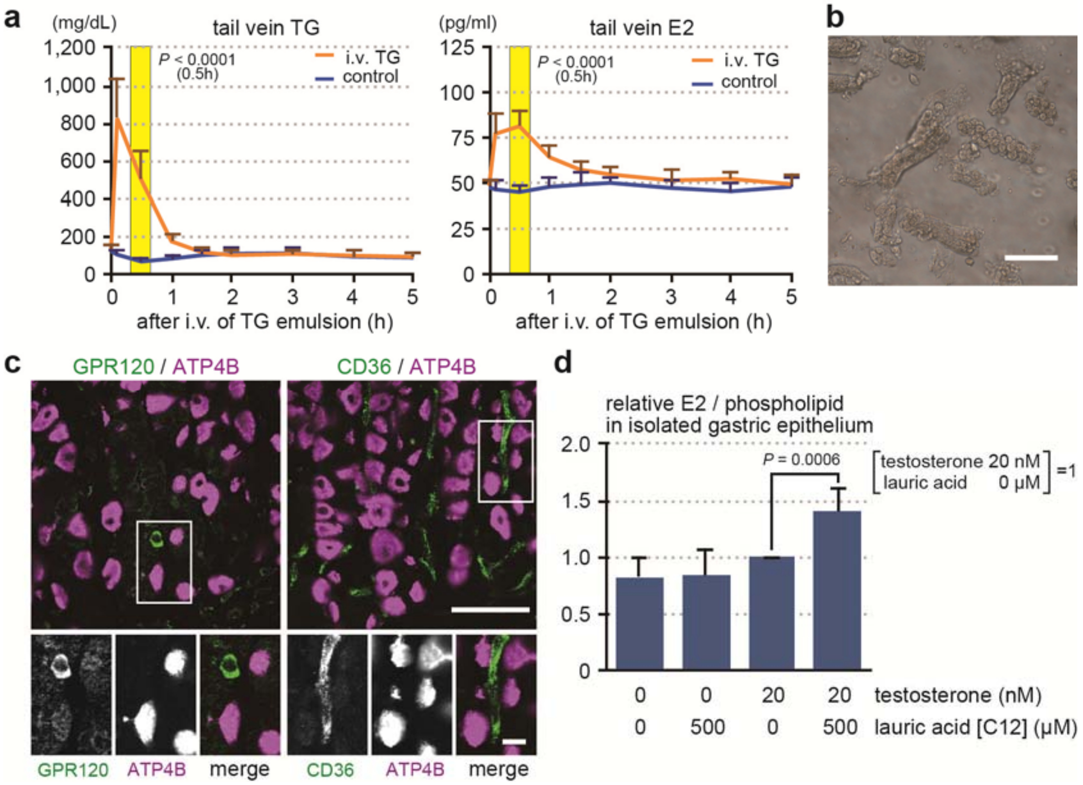

Fig. 4 Blood estrogen levels and production of gastric estrogen are directly regulated by blood triglyceride levels in males. a, Male rats (8-week-old, deprived of food for 4 h) were intravenously injected with soy oil emulsion (2 mL of 20 % soy oil emulsion per kg body weight, *n* = 10) or control saline (control, *n* = 10). TG and E2 levels in the tail venous blood were measured before (0) and at 0.1, 0.5, 1, 1.5, 2, 3, 4, 5 h after the injection. **b**, Isolated gastric gland epithelium from male rats. **c**, Gastric mucosa of male rat was double stained for GPR120 (G-protein-coupled receptor 120) or CD36 (cluster of differentiation 36) (green) with ATP4B (magenta). **d**, Isolated epithelium from male rat (deprived of food for 4 h) was incubated in DMEM with or without testosterone (20 nM) or lauric acid (500 μM) at 37 ºC for 1 h. E2 levels, normalized by phospholipid, were compared to those incubated with 20 nM testosterone but without lauric acid (*n* = 9). *n*, number of rats. Data are mean ± s.d. **a** (0.5 h) and **d**, *P* values determined by two-sided Student's *t*-test. Bars: (main panels) 50 μm; (insets) 10 μm.

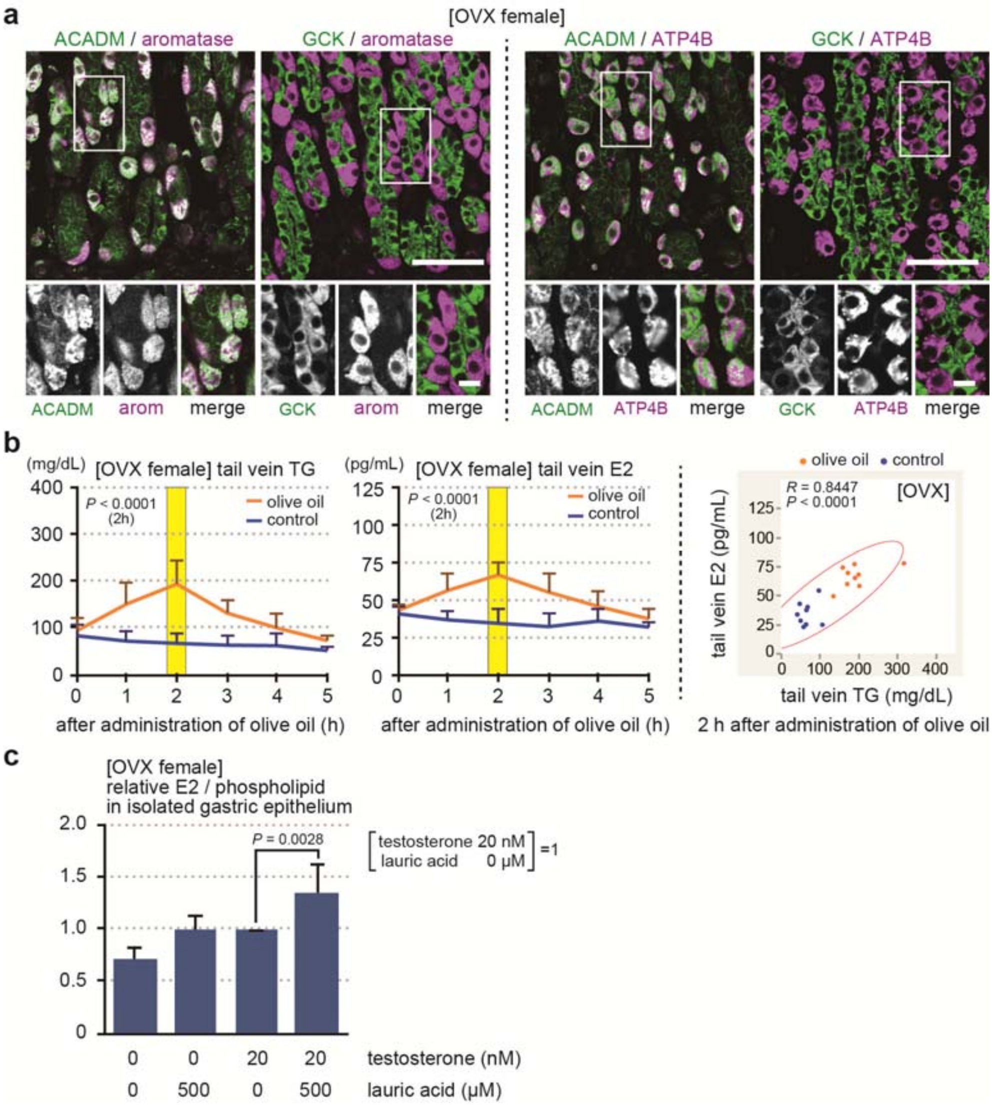

Fig. 5 Stomach of OVX female rats, like of male rats, secretes estrogen in response to the blood triglyceride levels. a, Gastric mucosa from OVX female rat (8-week-old, operated 14 days before) was double stained for ACADM or GCK (green) with aromatase [arom] or ATP4B (magenta). **b**, OVX female rats, deprived of food for 4 h, were orally administered olive oil (2.5 mL per kg body weight, n = 9) or control water (n = 9). TG and E2 levels in the tail venous blood measured before (0) and at 1, 2, 3, 4, 5 h after the administration (left), and the correlation diagram between the blood TG and E2 levels at 2 h after the administration (n = 18, right). **c**, Isolated epithelium from OVX female rat (deprived of food for 4 h) was incubated in DMEM with or without testosterone (20 nM) or lauric acid (500 μM) at 37 ºC for 1 h. E2 levels, normalized by phospholipid, were compared to those incubated with 20 nM testosterone but without lauric acid (n = 10). n, number of rats. Data are mean ± s.d. **b**, left (2 h) and **c**, P values determined by two-sided Student's t-test. **b**, right, R and P values determined by Pearson's product moment correlation with 95% density ellipse. Bars: (main panels) 50 μm; (insets) 10 μm.

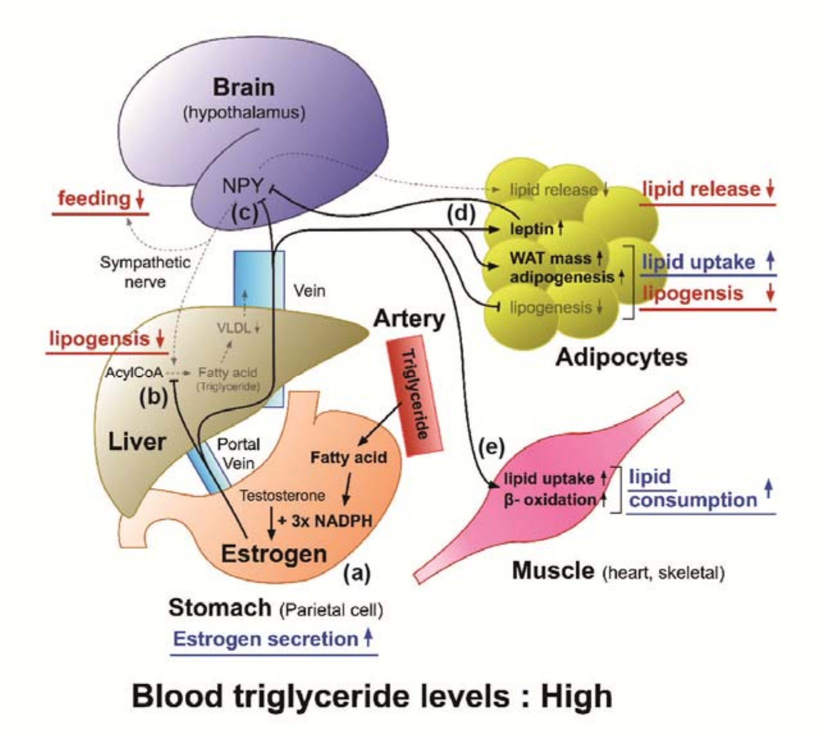

Fig. 6 Proposed model for the role of gastric estrogen in maintaining proper blood triglyceride levels. a, Parietal cells in the stomach produce estrogen using fatty acid (triglyceride) as an energy source (NADPH), and secretion of gastric estrogen increases as blood triglyceride levels rise (this study). **b**, Gastric estrogen, secreted into the portal vein, primarily acts on the liver to suppress hepatic *de novo* lipogenesis[15]. **c**, Gastric estrogen inhibits hypothalamic NPY neurons, whose activation enhances feeding behavior, hepatic *de novo* lipogenesis and fatty acid release from adipocytes via sympathetic nerves[19,44-47]. **d**, Gastric estrogen acts on adipocytes to suppress their *de novo* lipogenesis and to increase leptin secretion, white adipose tissue (WAT) mass and adipogenesis[17]. Leptin, in turn, inhibits hypothalamic NPY neurons[5]. **e**, Gastric estrogen increases the lipid uptake and β-oxidation of heart and skeletal muscles[20]. **a-e,** When blood triglyceride levels are high, secretion of gastric estrogen increases. Increased estrogen inhibits feeding behavior, *de novo* lipogenesis, and lipid release from adipose tissues, while promoting lipid uptake by WAT and lipid consumption by muscles. As a result, blood triglyceride levels are lowered. Conversely, when blood triglyceride levels are low, secretions of gastric estrogen are decreased to supply more lipid to the circulation.

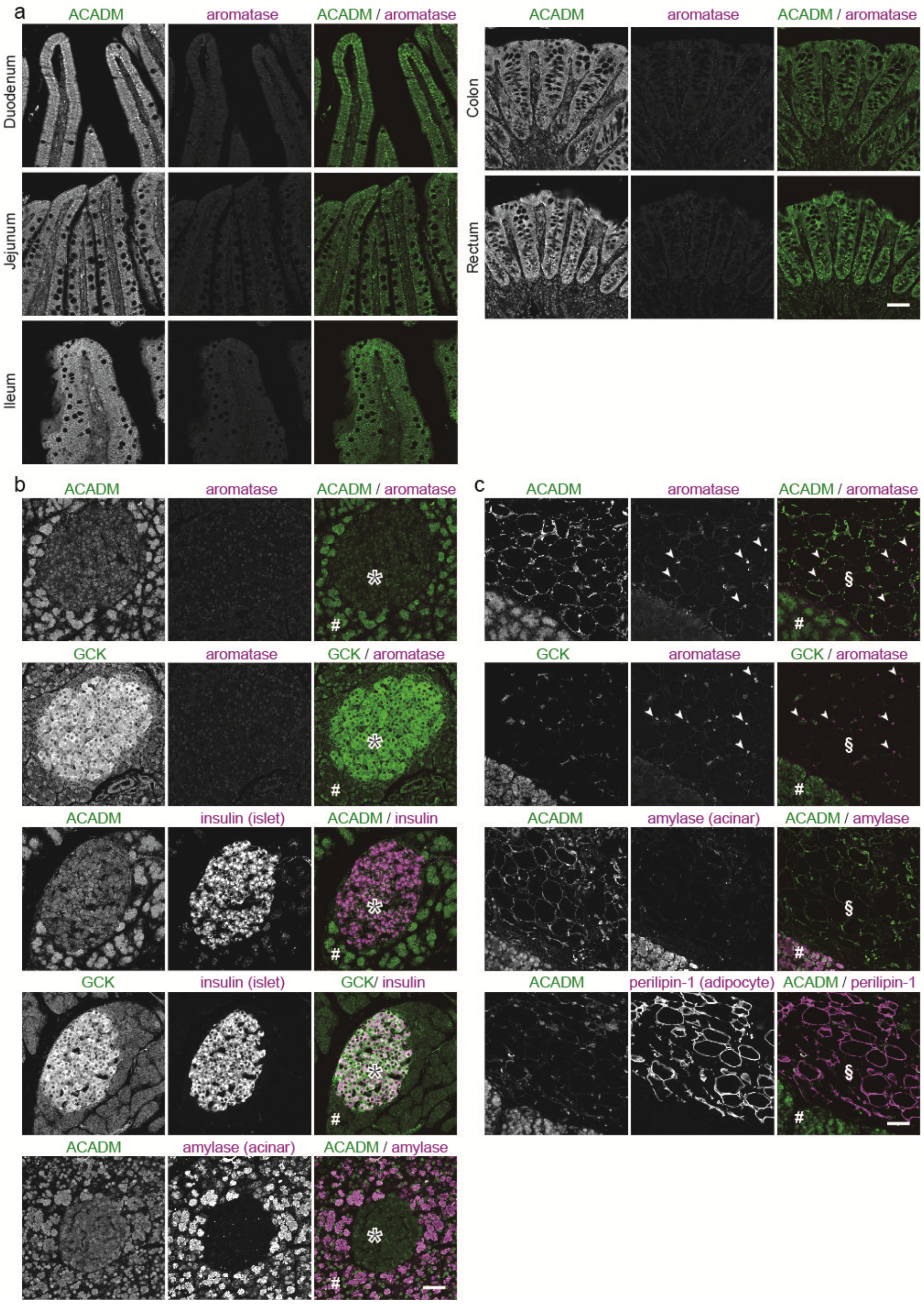

Supplementary Figure 1. Intestines and pancreas do not express aromatase, except for the attached adipocytes. **a**, Rat duodenums, jejunums, ileums, colons and rectums were double stained for ACADM (green) with aromatase (magenta). **b**, Central regions of rat pancreas were double stained for ACADM or GCK (green) with aromatase, insulin (islet marker; "*") or amylase (acinar cell marker; "#") (magenta). **c**, Peripheral regions of rat pancreas were double stained for ACADM or GCK (green) with aromatase, amylase (acinar cell marker; "#") or perilipin-1 (adipocyte marker; "§") (magenta). Arrowheads indicate the strong expression of aromatase in adipocytes. Bars: 50 µm.

REVIEWERS' COMMENTS:

Reviewer #1 (Remarks to the Author):

The MS has been revised as per my comments and I agree with the conclusions of the authors. i, therefore, recommend its publication.

Reviewer #2 (Remarks to the Author):

This revised manuscript responds to my previous comments and concerns well and is greatly improved. I have no further comments.
